# Accurate characterization of CRISPR-Cas9 genome editing outcomes and mosaicism with near-perfect long reads

**DOI:** 10.1186/s13073-026-01772-1

**Published:** 2026-09-15

**Authors:** Ida Höijer, Robin van Schendel, Anastasia Emmanouilidou, Rebecka Östlund, Ignas Bunikis, Marcel Tijsterman, Marcel den Hoed, Adam Ameur

**Affiliations:** 1 Department of Immunology, Genetics and Pathology, Uppsala University, Uppsala, Sweden; 2 SciLifeLab, Uppsala University, Uppsala, Sweden; 3 Department of Human Genetics, Leiden University Medical Center, Leiden, The Netherlands

**Keywords:** CRISPR-Cas9, Genome editing, Mosaicism, Off-target mutations, Structural variation, Long-read sequencing, Single-molecule sequencing, Targeted sequencing, PureTarget

## Abstract

**Background:**

Genetic mosaicism is a well-recognized consequence of CRISPR–Cas9 genome editing, yet its characterization remains challenging, especially when it involves low-frequency structural variants. A comprehensive analysis of mosaicism requires deep and unbiased sequencing of the target loci, with accurate single-molecule reads.

**Methods:**

We performed amplification-free PureTarget PacBio sequencing to investigate CRISPR-Cas9 outcomes at on-target and off-target sites in genome edited zebrafish and their offspring. CRISPR-Cas9 genome editing was performed by micro-injection in fertilized eggs at the single-cell stage.

**Results:**

Thirty samples from pooled larvae and individual zebrafish were successfully sequenced, resulting in > 1100x average target coverage. The PacBio reads reached an exceptional accuracy (QV39) over the target regions, with every read originating from a unique DNA molecule. The two haplotypes of the target loci displayed a balanced depth of coverage, while long-range PCR of the same samples resulted in skewed data. Further analysis of the PureTarget data revealed widespread genetic mosaicism in individual founder (F0) fish, with up to 18 distinct on-target events and 11 off-target events present in a single adult founder. Several CRISPR-Cas9 editing outcomes, including large structural variants and off-target mutations, were inherited to the F1 generation. Notably, as many as seven unique editing events were found among sibling F1 juvenile offspring derived from a single founder pair, thereby confirming the presence of genetic mosaicism in germ cells of founder zebrafish. This implies that some consequences of CRISPR-Cas9 editing may emerge only in the second generation. We also analyzed DNA methylation signals in the PureTarget data but did not observe altered 5mC CpG levels in genome edited samples.

**Conclusions:**

PureTarget enables efficient, accurate, and unbiased profiling of genetic mosaicism and DNA methylation at pre-defined genomic regions. Our results show that CRISPR–Cas9–induced mosaicism is widespread and represents an important factor to consider in genome editing experiments.

**Supplementary Information:**

The online version contains supplementary material available at https://doi.org/10.1186/s13073-026-01772-1.

## Background

Due to its efficiency and ease of use, CRISPR-Cas9 has become an indispensable tool for precise modification of the genome sequence in living cells and organisms. In a typical genome editing experiment, the CRISPR-Cas9 complex is directed to its target site by a guide RNA (gRNA) molecule. Once it has reached its target, Cas9 cleaves the DNA molecule with a double-strand break. The broken DNA is then repaired and this process can result in alterations of the DNA sequence at the cleavage site. In absence of a donor template, the DNA repair process often introduces small insertions and deletions, but to some extent also larger structural variants (SVs), and possibly even complex events such as whole chromosome deletions or chromothripsis [1–4]. Occasionally, the gRNA can bind to other locations than the intended (on-target) site, leading to off-target genome editing [5, 6]. Since it may be difficult to foresee the exact outcomes of a CRISPR-Cas9 experiment, the on-target and off-target regions are often verified by DNA sequencing to rule out the presence of undesired genome editing at the on-target and off-target sites.

In many cases, CRISPR-Cas9 generates multiple editing outcomes at the target site [7–9]. Consequently, a genome edited sample may contain a mosaic of small and large genetic variants, each of which could be either common or rare, and located at the on-target or at an off-target site. CRISPR-induced mosaicism has been observed in cell samples as well as in living organisms, where different edits may be present in different cell types or tissues. Genetic mosaicism has been found also in founder animals resulting from genome editing of fertilized eggs or embryos, and it is believed that the rate of mosaicism can be accelerated by the CRISPR-Cas9 complex remaining active through several cell divisions [10]. A recent study employing targeted amplicon sequencing in single cells revealed a unique editing pattern in nearly every cell [11]. However, in this particular study the cells were triple-edited, generating a significantly higher level of genetic heterogeneity and mosaicism as compared to a typical single-site editing experiment. The full extent of CRISPR-Cas9 induced genetic mosaicism occurring in a tissue sample, or in an organism, has not yet been possible to determine. New and improved sequencing methods are required to remove technical hurdles so this phenomenon can be studied in more detail.

The ideal sequencing approach for in-depth analysis of CRISPR-induced mosaicism needs to fulfill some key criteria. Firstly, the sequencing reads must be long enough so that not only small variants but also many of the larger SVs can be detected. Secondly, the data needs to have high quality so the exact variant breakpoints can be determined in individual reads. Thirdly, the method needs to yield high-coverage unique molecule data over the targets. For detection of low frequency variants down to 1% at least 200x coverage is required. Lastly, the sequencing data must be unbiased so that the identified variants represent the true distribution of editing outcomes occurring in the sample. The three first criteria can be met by long-range PCR (LR-PCR) and subsequent long-read sequencing, a method that can be further refined by unique molecular identifiers (UMIs) to remove duplicate molecules and increase the accuracy [12, 13]. However, even with UMIs, PCR amplification has intrinsic biases, particularly when amplifying longer regions. An amplification-free sequencing approach is therefore preferable.

Amplification-free sequencing can be achieved using instrumentation from Oxford Nanopore Technologies (ONT) or Pacific Biosciences (PacBio) [14]. Whole genome long-read sequencing (WG-LRS) gives an unbiased and complete representation of the genome. However, it is not suitable for studying mosaicism since the cost of generating > 200X coverage WG-LRS data is prohibitively high when investigating many samples. A more feasible option is ONT’s adaptive sampling, where target molecules are selected during sequencing [15, 16]. Adaptive sampling has been seen to generate 9x to 40x median target coverage, or 4.6-fold enrichment when run on a MinION flow cell [17]. This means that at most a handful of samples can be multiplexed on a PromethION flow cell in order to reach sufficient target coverage for mosaicism analysis, an experimental setup that still would be relatively costly. Furthermore, ONT sequencing has limitations in indel calling [18], potentially rendering it suboptimal for analysis of mosaicism in CRISPR-Cas9 edited samples, which often consist of small insertions and deletions.

An alternative approach capable of generating deeper coverage over the targets is Cas9-based enrichment in combination with long-read sequencing [19]. Such protocols have been developed both for ONT and PacBio, primarily with the aim of resolving repeat expansions in neurological disorders [20, 21]. ONT’s Cas9 enrichment has been applied for genome editing verification with promising results, although the error rate limits the usefulness of the method and complicates the analysis [22, 23]. PacBio recently released the PureTarget Cas9-enrichment method along with a panel of gRNAs to target 38 disease causing repeat loci in the human genome. The workflow allows multiplexing of up to 48 samples on a single Revio SMRTcell. In PacBio sequencing, the molecules are circularized by ligating hairpin adaptors, and each base is sequenced several times to generate very high accuracy (HiFi) data over the target regions [24]. With PureTarget, the enriched DNA is shorter than the 15–20 kb length in a typical PacBio HiFi library, thereby enabling additional passes of the circular template during sequencing, which could further increase the accuracy. Thus, we reasoned that PureTarget, due to its specific properties, might be able to generate single-molecule reads with sufficient target coverage and quality for detailed investigation of genetic mosaicism in CRISPR-Cas9 genome edited samples.

Long-read sequencing technologies not only provide DNA sequence information but also signals for base modifications like CpG 5mC methylation [25, 26]. At present, little is known about to what extent 5mC modifications can be affected by CRISPR-Cas9 genome editing. However, a recent report examined the effect of CRISPR-Cas9 induced double-strand breaks in differentially methylated regions of imprinted loci [27]. Using targeted ONT sequencing, the authors detected significant DNA methylation alterations in genome edited samples, likely caused by large structural variants, homologous recombination, or defective methylation maintenance during DNA repair. It is plausible that CRISPR-Cas9 can induce methylation changes also outside of imprinted regions, for example by interfering with the regulatory function of enhancers and promoters. Thus, the ability to include DNA methylation data in the assessment of CRISPR-Cas9 genome editing outcomes may prove important for future studies, since it could be indicative of alterations in gene expression.

Here, we applied PureTarget to study genetic outcomes of CRISPR-Cas9 editing. The samples were previously examined by us using LR-PCR as part of a study published in 2022, where off-target genome editing and large structural variant events were studied in two generations of zebrafish [4]. In the present study, we revisit the zebrafish DNA samples and evaluate PureTarget for simultaneous validation of genetic and epigenetic (5mC) variation induced by CRISPR-Cas9, with the aim to investigate mosaicism at an unprecedented level of detail.

## Methods

### Study cohort and zebrafish handling

Briefly, all zebrafish experiments and husbandry were conducted in accordance with Swedish and European regulations and have been approved by the Uppsala University Ethical Committee for Animal Research (Dnr 5.8.18–13680/2020). Thirty-two zebrafish samples consisting of 10 adult fish, 9 fin clips from juvenile fish and 385 larvae were analyzed in this study. The larvae were analyzed in pools consisting of 25 or 30 larvae. The adult fish and fin clips from juvenile fish were analyzed as individual samples. Complete information about the study cohort is found in Additional file 1: Table S1.

### CRISPR-Cas9 genome editing and sample collection

The CRISPR-Cas9 genome editing of zebrafish was performed for an earlier study by Höijer et al. [4]. The genes of interest were targeted using one gRNA (Additional file 1: Table S2) per orthologue with an anticipated editing efficiency > 90%. The gRNAs were designed to target an early exon of the genes. RNA duplexes of the chemically synthesized Alt-RcrRNA (IDT) and Alt-R^®^ tracrRNA (IDT) were complexed with Alt-R^®^ S.p. Cas9 nuclease, v.3 (IDT) to form “duplex guide RNPs” (dgRNPs), as described by Hoshijima K et al. [28]. The dgRNPs were then injected into fertilized zebrafish eggs at the 1-cell stage. Uninjected embryos were kept and used as controls. The injected founder embryos were raised to adulthood at which time randomly selected mating pairs were in-crossed. Pools of 25–30 10-day-old F0 larvae, individual F0 adult fish, pools of 30 5-day-old F1 larvae, and fin clips of individual juvenile F1 fish were collected throughout the experiment for downstream analyses (Additional file 1: Table S1). All sample types were snap frozen in liquid nitrogen upon collection, to ensure DNA integrity. Adult zebrafish were sacrificed by prolonged exposure to tricaine prior to the snap freezing step.

### Extraction of genomic DNA from zebrafish

All samples were extracted using the MagAttract HMW DNA Kit (Qiagen) and the “Manual Purification of High-Molecular-Weight Genomic DNA from Fresh or Frozen Tissue” protocol according to the manufacturer’s instructions. A tissue homogenization step using a pestle was added to the protocol prior to the lysis step. DNA integrity of the extracted samples was assessed using the Femto Pulse system (Agilent Technologies) using the Genomic DNA 165 kb kit.

### Amplicon construction and sequencing

LR-PCR and sequencing was performed in an earlier study for 31 of the 32 samples (Additional file 1: Table S1) [4], as follows: Primers were designed to span on-target sites for *ldlra*, *nbeal2*, *sh2b3* and *ywhaqa* and one off-target site for *sh2b3* and two off-target sites for *ywhaqa*. The amplified regions ranged from 4.8 kb to 7.7 kb in size. Primer sequences, expected amplicon sizes, and primer coordinates are found in Additional file 1: Table S3 and Additional file 1: Fig. S1. Long-range PCRs were performed using the PrimeStar GLX Polymerase (Takara Bio) according to the manufacturer’s instructions, using either the standard or two-step PCR cycling protocol (25–30 cycles). 0.2 µg/µl bovine serum albumin (BSA) was added to the PCR reactions for improved performance. 30 ng of genomic DNA from pooled or individual zebrafish DNA extractions was used for the PCRs. Amplicons originating from different primer pairs were pooled equimolarly. Amplicon pools were barcoded and sequenced on PacBio’s Sequel system using the SMRTbell^®^ Express Template Prep Kit 2.0 and the PacBio Barcoded Overhang Adapter Kit 8 A and 8B for SMRTbell construction, and 3.0 sequencing and binding chemistry using 10 h movie time.

### Design of excision gRNAs for PureTarget

Guide RNAs (gRNAs) for target excision were designed using CHOPCHOP v.3 [29] and were ordered as Alt-R™ CRISPR-Cas9 sgRNA from Integrated DNA Technologies (IDT). For each region of interest (ROI) two gRNAs were designed, one upstream and one downstream of the ROI. The gRNAs were designed to enrich for the same regions as the LR-PCR primers, i.e. on-target sites for *ldlra*, *nbeal2*,* sh2b3* and *ywhaqa* and one off-target site for *sh2b3* and two off-target sites for *ywhaqa.* The size of the enriched targets was designed to be approximately 5 kb in size. Upon arrival the gRNAs were pooled equimolarly to 5 µM and stored for future usage. Further information about the gRNAs is available in Additional file 1: Table S4 and Additional file 1: Fig. S1.

### PureTarget enrichment of on and off-target sites and PacBio sequencing

Genomic DNA from 32 zebrafish samples was used to prepare PureTarget libraries. PacBio recommends that at least 50% of DNA fragments are > 30 kb (GQN 30 kb). However, samples that did not meet this criterion were nevertheless included in the library preparation (Additional file 1: Table S5). 1.5 µg DNA from each of the 32 zebrafish samples was initially processed in a gDNA repair step using a R&D protocol provided by PacBio. In this step, 5 µl of repair buffer (PacBio) and 1 µl of DNA repair mix (PacBio) were added to each sample and incubated at 37 °C for 60 min, followed by 52 °C for 30 min. Subsequently, the samples were bead-washed using 1x of SMRTbell cleanup beads (PacBio). Following gDNA repair, the 32 samples were processed using the PureTarget repeat expansion panel kit and the “Generating PureTarget repeat expansion panel libraries” protocol (PacBio) according to manufacturer’s instructions. We used our own gRNA pool instead of the repeat expansion panel provided with the kit. During library preparation all 32 samples were multiplexed and sequenced as one pool on one PacBio Revio SMRTcell, using SPRQ chemistry and 24 h movie time. SMRT Link v. 25.2 was used for sequencing and primary data processing.

### Alignment of PacBio reads and data analysis overview

The reads were aligned to the zebrafish reference genome (GRCz11) using the “spliced” parameter in minimap2 v.2.26 [30]. This parameter is mainly intended for alignment of long RNA data, but we found it to improve alignment for large deletions in the target regions, both for the PureTarget and LR-PCR data. However, since the minimap2 “spliced” alignment did not preserve the 5mC methylation signals, we performed a separate alignment using SMRTLink v25.2 with default settings for the PureTarget data, using default parameters. This yielded alignment files that include 5mC methylation signals. These files were uploaded to the European Nucleotide Archive (ENA) to ensure that 5mC information was preserved in the archived data (accession number PRJEB110416). The “spliced” alignment files were used for the coverage analysis, quality assessment enrichment rate analysis described below. Visualization of the aligned reads was done using IGV v 2.19.4 [31]. Detection and visualization of the CRISPR-Cas9 induced genome editing events was performed using the Sequence Interrogation and Qualification SIQ (SIQ) software [32], which does not rely on the alignment files described in this section.

### PureTarget coverage analysis and quality assessment

Two of the zebrafish samples had less than 30X coverage for all target regions and were removed from further analysis. The two samples that failed were among those having the lowest Genomic Quality Number (GQN) at 30 kb, suggesting the failure was at least partly caused by poor DNA quality (Additional file 1: Table S5). For the remaining 30 samples, the average coverage across all sites was obtained by applying the ‘coverage’ function in samtools v1.20 [33] to the data aligned to the regions of interest. To calculate quality scores, fastq files were extracted for each of the sites using samtools ‘bam2fq’ and FastQC v0.11.9 (www.bioinformatics.babraham.ac.uk/projects/fastqc*)* was used to generate phred quality score distributions for each sample and target.

### Calculating the PureTarget enrichment rate

The GRCz11 chromosomal sequences consist of 1.345 Gb and the combined size of the seven target regions in the PureTarget design is 33,258 bp. This means that the targets cover about 0.0025% of the zebrafish reference genome. In total, the PureTarget sequencing generated 10.4 Gb HiFi data from one Revio SMRTcell. If sequencing was completely random, we expect a mean coverage of 0.243x for each of the 32 samples over target regions. However, in the PureTarget experiment we reach a mean target coverage of 1096x for the 32 samples across the seven on- and off-target regions. This implies that PureTarget has an enrichment rate of about 4500x as compared with shotgun whole genome sequencing. When removing the two samples that generated less than 30x coverage for all target regions, the mean target coverage was 1168x. This corresponds to an enrichment rate of 4800x.

### Detection of and visualization of genome editing events using SIQ

Analysis of CRISPR-Cas9 genome editing outcomes was performed using SIQ v2.1 [32] and the results were visualized using SIQplotteR [32] As input to SIQ, we used fastq files extracted from each mapped bam file for each sample/target combination. The analysis was run both for the PureTarget and LR-PCR data. To minimize the risk of detecting false positive events, i.e. naturally occurring genetic variation instead of true CRISPR-Cas9 induced variants, only events occurring within 2 bp of the expected CRISPR-Cas9 genome editing site were considered in the SIQ analysis. A cut-off of 1% was applied for the detection of mosaic events in a sample.

## Results

### High-quality long-read sequencing of CRISPR-Cas9 genome editing outcomes using PureTarget

**Fig. 1:**
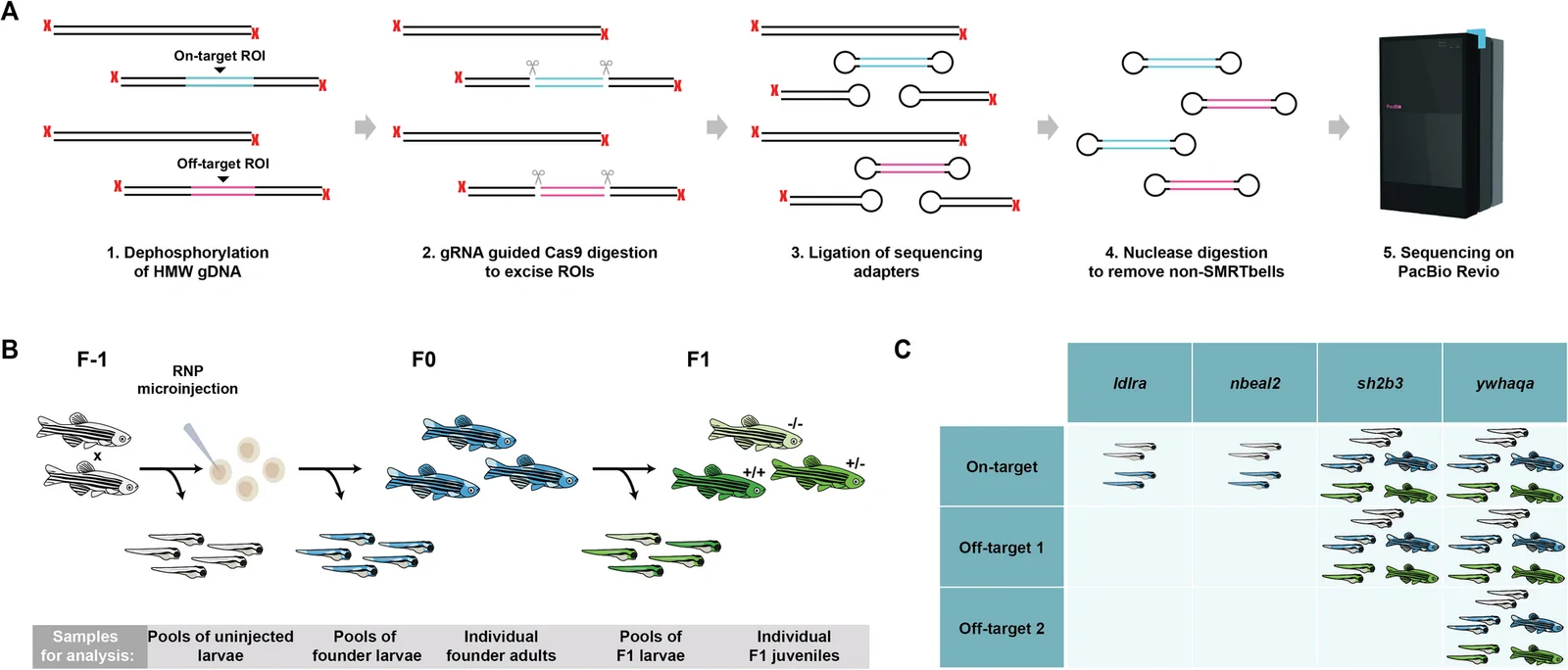
Overview of the targeted sequencing method and samples used in the study. **(A)** The steps of the PureTarget protocol for Cas9-based target enrichment and PacBio sequencing. **(B)** Overview of the zebrafish experiment. CRISPR-Cas9 genome editing was performed by micro-injection in fertilized eggs at the single-cell stage. Samples were obtained from pooled larvae and individual zebrafish from the founder (F0) and F1 generations. **(C)** Four gRNAs previously shown to induce off-target mutagenesis were used for CRISPR-Cas9 gene editing of *ldlra*, *nbeal2*, *sh2b3* and *ywhaqa*. The table shows the fish samples that were assayed at the on- and off-target sites for the four different gRNAs. In the table, the larvae and fish are color-coded according to the experimental outline in panel B with unedited samples (in gray), edited F0 samples (blue) and edited F1 samples (green)

PureTarget is an amplification-free protocol for targeted PacBio sequencing, initially developed for re-sequencing of repeat expansions in human genomes. It is a Cas9-based enrichment method, where Cas9 nuclease is guided by two target-specific gRNAs to excise a region of interest (ROI). Here, we adapted PureTarget to investigate genome editing outcomes (Fig. 1A) and applied it to DNA samples from two generations of CRISPR-Cas9 edited zebrafish previously analyzed by long-range PCR (LR-PCR) and PacBio sequencing [4] (Fig. 1B). Four on-target genome editing sites were investigated in the zebrafish samples, targeting coding sequence in the genes *ldlra*, *nbeal2*, *sh2b3* and *ywhaqa* (Additional file 1: Table S2). Three previously identified off-target sites were examined as well, one for *sh2b3* and two for *ywhaqa* (Fig. 1C and Additional file 1: Table S4). The ROIs were designed to have a length of approximately 5 kb (Additional file 1: Fig. S1). The 32 samples consisted of six pools of 25–30 10-day-old edited founder larvae, six pools of 30 5-day-old edited F1 larvae, ten individual edited adult founder fish, nine individual edited juvenile F1 fish, and one pool of unedited founder larvae serving as a control sample (Additional file 1: Table S5). The samples were partially degraded (Additional file 1: Fig. S2 and Additional file 1: Table S5) due to difficulties to extract intact DNA from zebrafish. Despite this, PacBio sequencing of the 32 PureTarget samples on a single Revio 8 M SMRTcell generated 1.8 million reads (Additional file 1: Table S6). 17.1% of reads were aligned to the ROIs, corresponding to an average enrichment rate of 4500x for target regions vs. the rest of the zebrafish genome (Additional file 1: Table S7 and Methods). The PacBio HiFi reads that were aligned to the ROIs had an average QV score of 39 (Additional file 1: Fig. S3). Although not an exact measure of quality, QV39 corresponds to one error in 7900 bases, implying that many reads mapping to ROIs are completely error-free. The PureTarget assay thus enables single-molecule sequencing of the CRISPR-Cas9 editing sites with near-perfect kilobase-length reads.

### Unbiased and in-depth analysis of on-target and off-target editing sites in zebrafish

**Fig. 2:**
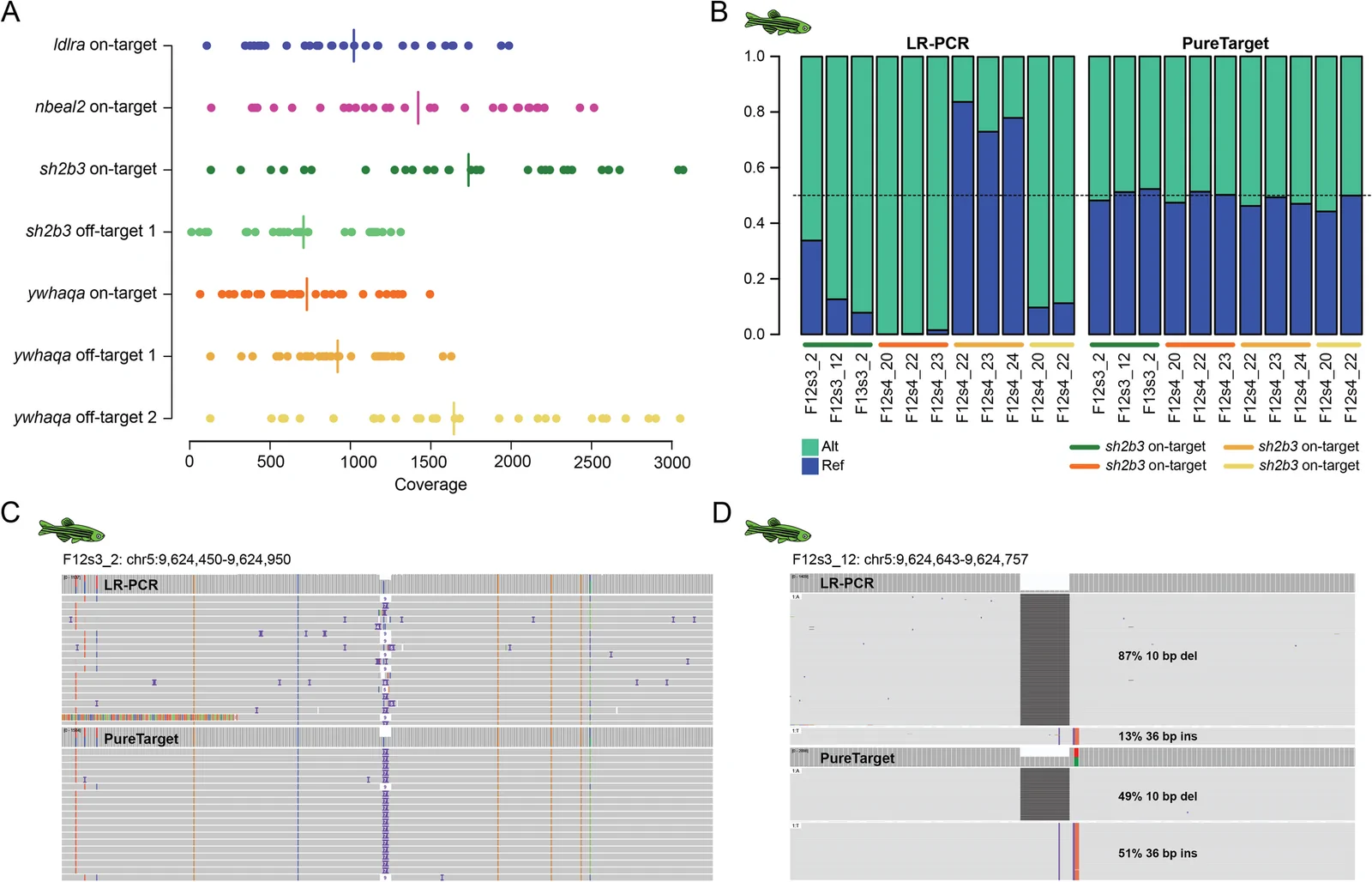
Performance of the PureTarget method on genome edited zebrafish samples. **(A)** Coverage across the 30 PureTarget zebrafish samples and seven target regions included in the assay. The vertical lines correspond to the average coverage for the different targets. **(B)** Frequencies of the reference (blue) and alternative (green) alleles at single nucleotide variant (SNV) positions in heterozygous target regions for F1 zebrafish. The bars to the left show allele frequencies for long-range PCR (LR-PCR) while the bars to the right show allele frequencies for PureTarget in the same samples. The minor allele frequencies obtained by PureTarget are close to 50% while LR-PCR results in more uneven distribution between the two haplotypes. The colors below the bars indicate which of the target sites from A were examined. **(C)** IGV visualization of the *sh2b3* on-target region in an F1 juvenile fish, centered around the CRISPR-Cas9 genome editing site. The IGV tracks show representative PacBio read alignments for LR-PCR (top) and PureTarget (bottom), although not all reads are displayed in this view. The PureTarget data contains fewer alignment errors and displays a clearer view of the editing outcomes. **(D)** Visualization of the *sh2b3* on-target region in an F1 juvenile fish showing results for LR-PCR (top) and PureTarget (bottom). The view has been compressed so all reads are shown for each sample. The sample contains two CRISPR-Cas9 editing outcomes, a 10 bp deletion and a 36 bp insertion. In the LR-PCR data the 10 bp deletion is found in 87% of the reads. For PureTarget, the two alleles have an even balance with 49% of the reads containing the deletion and 51% containing the insertion

Two samples from adult founder fish generated low (< 30x) coverage of the targeted regions, because of suboptimal Cas9 enrichment or poor DNA quality. These two samples were excluded from the downstream analysis. The 30 remaining samples reached 1168x coverage on average at the seven ROIs, with some samples having > 3000x coverage at specific targets (Fig. 2A). We were further interested to evaluate the coverage uniformity for the two haplotypes in individual zebrafish. For this purpose, we analyzed the allelic distribution at heterozygous SNVs in zebrafish from the F1 generation. We did not include founder fish in this analysis because they may have a skewed allele frequency due to loss of heterozygosity and mosaicism induced by CRISPR-Cas9 editing. In total, eleven heterozygous on-target and off-target regions were found in seven individual F1 fish. A representative SNV position was selected in each target as a measure of allelic distribution. We then compared the allele frequencies at the SNV position in the PureTarget results to LR-PCR data from the same samples (Additional file 1: Table S8). Our results show that PureTarget gives a near 50–50% distribution between the reference and alternative alleles, also in cases where LR-PCR is highly biased (Fig. 2B). The LR-PCR allele frequencies differed between samples and amplicon regions, and the observed biases were likely caused by an interplay of several factors. For one F1 zebrafish, a heterozygous 74 bp AT repeat in the *ywhaqa* on-target ROI was found to inhibit PCR amplification (Additional file 1: Fig. S4). In other cases, the bias may be due to structural variants that generate length differences between the haplotypes and preferential amplification of the shorter allele. The biases can be further aggravated by sub-optimal LR-PCR conditions, such as low amounts of starting material or an increased number of PCR cycles. Viewing the data in IGV [31] demonstrates the advantages of PureTarget when it comes to generating high-quality reads and equal representation of the two haplotypes (Fig. 2C-D). The exceptional quality of the PureTarget reads alongside its unbiased nature makes the method ideal to investigate low-frequency and mosaic events in genome edited samples.

### Founder zebrafish are mosaic at on- and off-target CRISPR-Cas9 editing sites

**Fig. 3:**
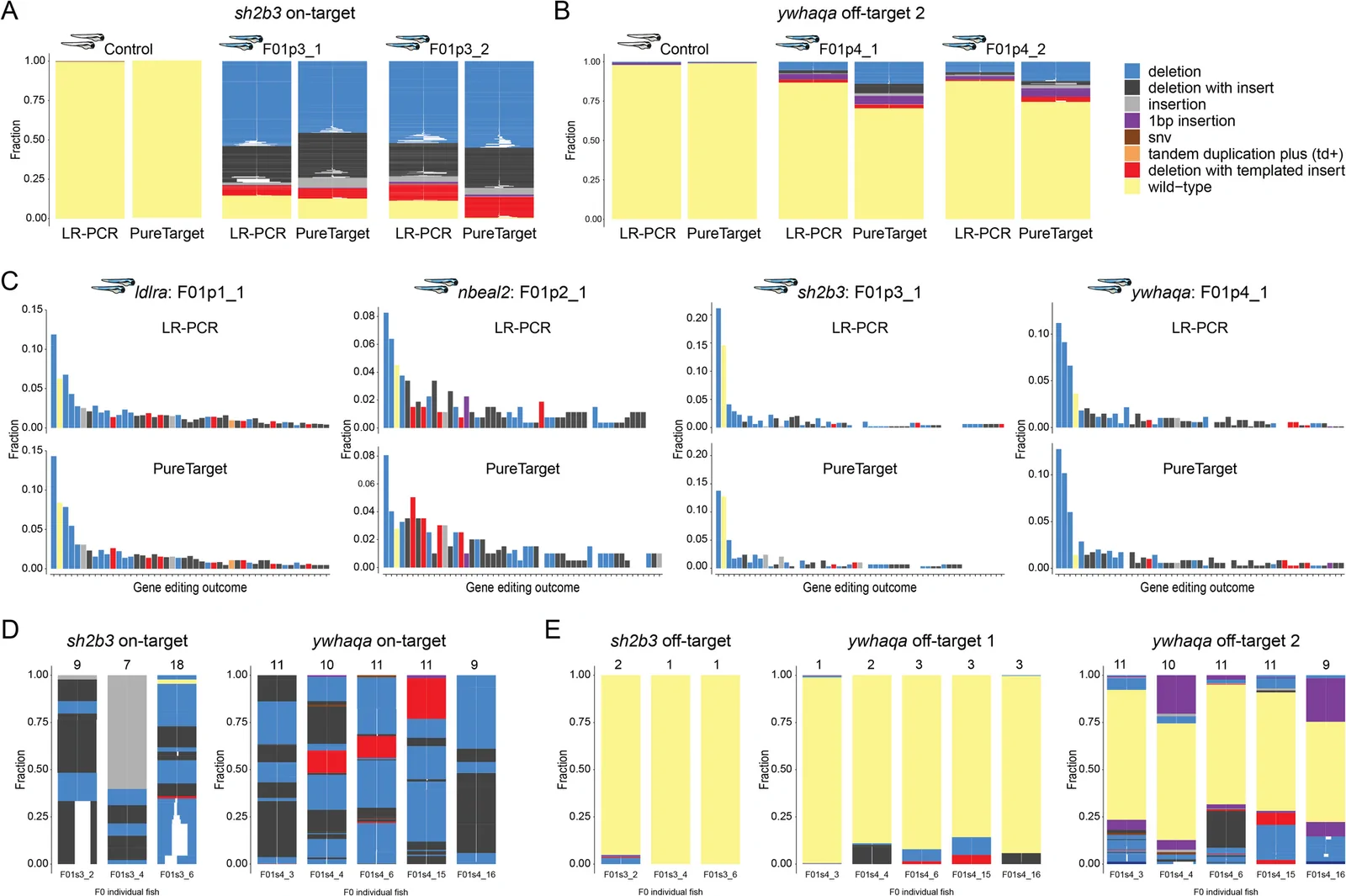
CRISPR-Cas9 induced genetic mosaicism in founder zebrafish. **(A)** SIQ tornado plots showing the distribution of on-target genome editing outcomes in the uninjected control sample and two pools of *sh2b3* founder (F0) larvae. For each sample, outcomes are shown both for LR-PCR and PureTarget data in a ± 2 kb window centered around the CRISPR-Cas9 editing site. The pools of larvae contain many distinct genome editing outcomes while only the wild-type allele is detected in the control sample. **(B)** SIQ tornado plots for uninjected control sample and two pools of *ywhaqa* founder (F0) larvae at *ywhaqa* off-target site 2 (1 kb window size). **(C)** Comparison of CRISPR-Cas9 genome editing outcome frequencies in *ldlra*, *nbeal2*, *sh2b3* and *ywhaqa* in pooled founder larvae. For each sample of pooled founder larvae, a comparison is shown between LR-PCR (top) and PureTarget (bottom). Each plot shows the ~ 50 highest frequency editing outcomes. There is a good overlap between events detected by LR-PCR and PureTarget, but with varying frequencies for some events. **(D)** SIQ visualization of all editing events detected at *sh2b3* and *ywhaqa* on-targets in adult founder individuals. At the top of each bar are the number of distinct events with allele frequency > 1%. **(E)** Editing events at off-target sites for the same founder individuals as in D

Having verified the sensitivity of our method for targeted sequencing of zebrafish DNA, we were interested to investigate the level of mosaicism occurring in the founder (F0) generation. For this purpose, we analyzed the PureTarget data using the software SIQ for analysis and SIQplotteR for visualization [32]. We compared the mutational outcomes in samples of pooled founder larvae analyzed both by PureTarget and LR-PCR. This revealed a wide range of editing events at the on-target sites, while – as expected – no editing was seen in the control sample (Fig. 3A and Additional file 1: Fig. S5). Editing was also observed at off-target sites, with the highest degree occurring at *ywhaqa* off-target 2 (Fig. 3B and Additional file 1: Fig. S6). In the samples of pooled founder larvae there was a large overlap between the on-target events identified by PureTarget and LR-PCR, although the allele frequency distributions showed a certain degree of variability (Fig. 3C). The discrepancies in allele frequencies are likely caused by biases in the LR-PCR data. We next examined the PureTarget data for individual adult founder zebrafish. The SIQ results revealed a high degree of on-target mosaicism for *sh2b3* and *ywhaqa*, with between 7 and 18 distinct events present in the founder individuals (Fig. 3D). These numbers were obtained by applying a 1% variant allele frequency (VAF) cut-off. Theoretical calculations indicate that 299x coverage is sufficient to detect variants at 1% VAF with 95% confidence (Additional file 1: Fig. S7), a threshold far exceeded by the enrichment achieved in our PureTarget assay. As expected, only the wild-type allele reached the 1% threshold for the control sample. Mosaicism was identified also at off-target sites, in particular for *ywhaqa* off-target 2, where 9–11 distinct alleles were seen in the adult individuals (Fig. 3E). Based on these results, we conclude that genetic mosaicism is highly abundant in adult founder zebrafish.

### CRISPR-Cas9 induced mosaicism is present also in germ cells of founder zebrafish

**Fig. 4:**
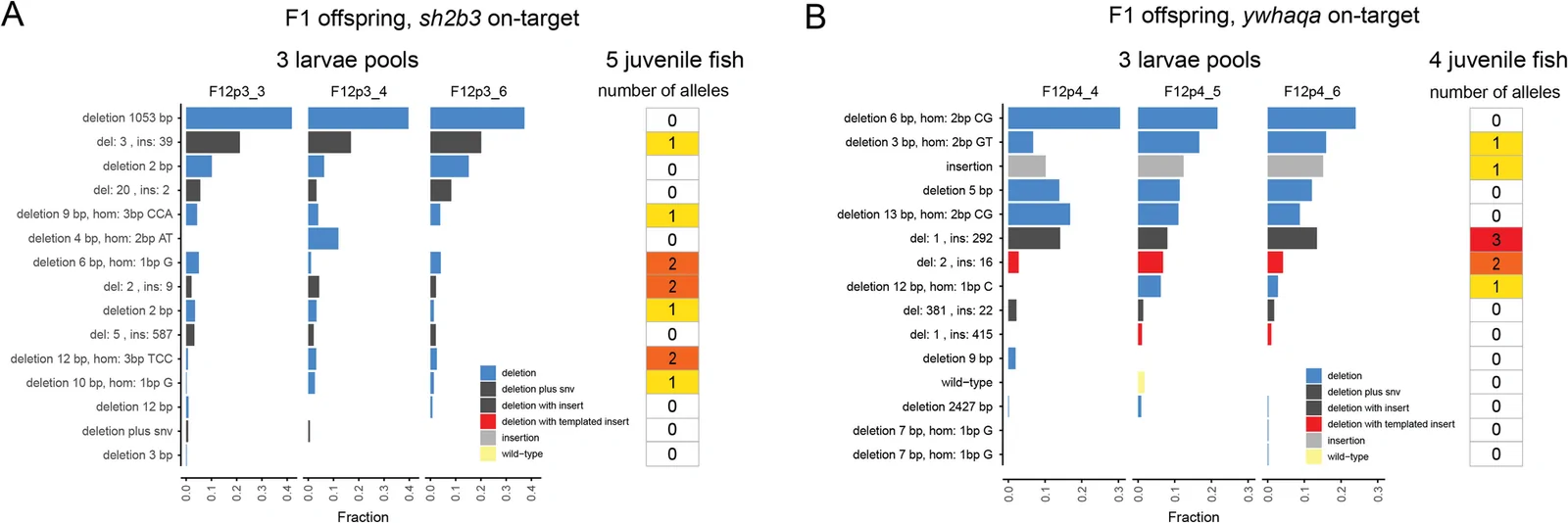
Overview of CRISPR-Cas9 induced on-target events in F1 offspring from the same founder pair. **(A)** SIQ image with allele frequencies for the three pools of *sh2b3*-edited F1 larvae displayed as horizontal bars. The top 15 most frequent events in the F1 larvae are shown in the plot. The column at the right shows the number of occurrences of the editing events among five juvenile fish. **(B)** SIQ image with allele frequencies for the three pools of *ywhaqa*-edited F1 larvae displayed as horizontal bars (at the left) and alleles from four individual juvenile F1 zebrafish (at the right). The 15 most frequent events in the *ywhaqa* F1 larvae pools are shown in the plot

We observed a wide range of genome-editing events in the founder zebrafish and examined how many of these were passed on to the next generation. In our experiment, we sequenced six samples of 30 pooled F1 larvae as well as nine samples from fin-clips of individual juvenile F1 zebrafish. The samples were collected from the offspring of two founder pair crossings, one for *sh2b3* and one for *ywhaqa*. From the offspring of the *sh2b3-*edited founder pair we generated PureTarget data for three F1 larvae pools and for five juvenile F1 fish. Three F1 larvae pools were sequenced also from the *ywhaqa*-edited founder pair offspring, and four juvenile F1 fish. For *sh2b3*, seven on-target editing events were present in all three samples of pooled larvae and in ≥ 1 of the juveniles (Fig. 4A). For *ywhaqa*, five on-target events were overlapping between all three larvae pools and at least one juvenile F1 fish (Fig. 4B). For the two investigated founder pairs, we thus found 5 and 7 unique on-target CRISPR-Cas9 editing outcomes among their juvenile F1 offspring. This observation can only be explained by the presence of mosaicism in the germ cells of the founder zebrafish. Several of the events detected in multiple pools of larvae were not seen in the juvenile F1 zebrafish. The absence of these events could be explained by the low sample size of F1 juveniles in our experiment and/or by a selective disadvantage of certain mutations during zebrafish development.

### Unintended genome editing outcomes are detectable in the F1 generation

**Fig. 5:**
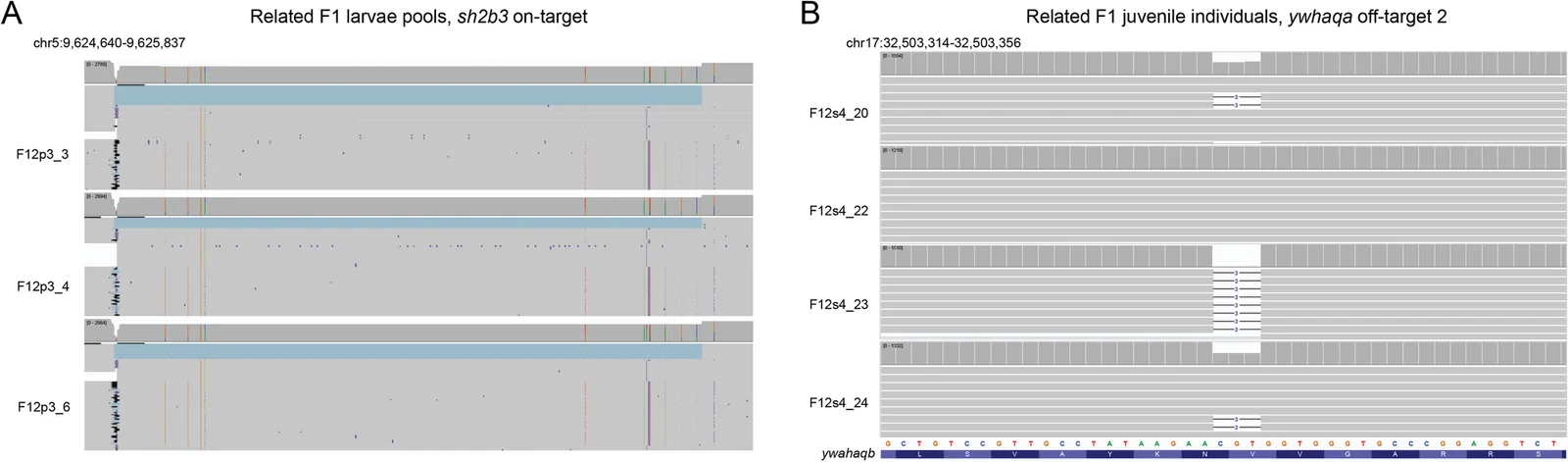
Examples of unintended genome editing events in the F1 generation. **(A)** IGV view of a 1053 bp deletion detected in pooled *sh2b3*-edited F1 larvae. The large deletion is represented by the light blue region that is present in all three samples of pooled larvae. **(B)** A three base pair deletion in the *ywhaqa* off-target 2 region, located inside an exon of the *ywhaqa* paralogue *ywhaqb*. The IGV view shows the presence of this off-target mutation with separate tracks for four juvenile F1 zebrafish. Two of the F1 fish are heterozygous, one F1 fish is homozygous for the off-target deletion, and one F1 fish is homozygous for the reference allele

We next analyzed “unintended” outcomes of CRISPR-Cas9 genome editing in the F1 zebrafish. For this reason, we focused on large on-target structural variants and off-target mutations previously detected in the LR-PCR data [4] and aimed to verify their presence in the PureTarget data. In all three samples of pooled *sh2b3*-edited F1 larvae, a deletion of 1053 bp was detected, an event that is clearly visible in IGV (Fig. 5A). This large deletion was the most frequent event in the SIQ analysis of F1 larvae (Fig. 4A) but it was not detected in any of the juvenile *sh2b3*-edited F1 zebrafish. One example of off-target genome editing was detected at *ywhaqa* off-target 2. This off-target deletion, which spanned 3 bp and was located in an exon of *ywhaqb*, was found to be homozygous in one juvenile fish and heterozygous in two other juveniles (Fig. 5B). The same 3 bp deletion in *ywhaqb* was present in all three pools of *ywhaqa*-edited F1 larvae. Our examples thus show that unexpected CRISPR-Cas9 editing outcomes, such as large on- and off-target deletions, can transfer to the next generation. As mentioned above, these examples were earlier using LR-PCR but we here corroborate the findings with PureTarget as an alternative sequencing method.

### CRISPR-Cas9 genome editing has no visible effect on 5mC CpG methylation

**Fig. 6:**
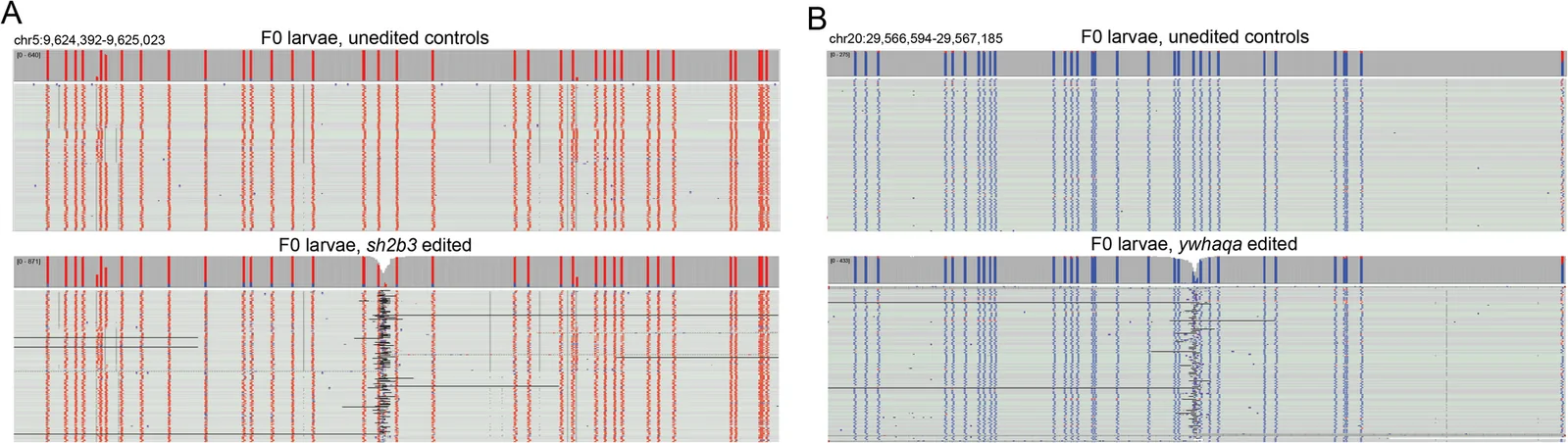
Comparison of 5mC CpG methylation signals between CRISPR-Cas9 edited and control samples. **(A)** IGV view showing 5mC signals in the PureTarget data from a sample of pooled *sh2b3* founder larvae (bottom) compared with unedited controls (top). The red lines indicate that the CpG signals in this region are methylated both in the *sh2b3*-edited larvae and unedited controls. **(B)** Methylation signals in pooled larvae edited at *ywhaqa* (bottom) compared with unedited controls (top). The blue lines indicate that the CpG signals in this region are unmethylated both in the edited larvae and controls

Since PureTarget is an amplification-free protocol for PacBio sequencing, it enables detection of CpG 5mC methylation signals in individual reads and quantification of allele-specific methylation [34]. We therefore aimed to investigate whether CRISPR-Cas9 genome editing had a noticeable effect on 5mC methylation signals in our PureTarget experiment. Hence, we compared the methylation patterns in pooled CRISPR-Cas9 edited founder larvae to the signals in the control sample. This analysis was performed only for the on-target regions where CRISPR-Cas9 editing was abundant. By visualizing the data in IGV, we could clearly see that the *sh2b3* on-target region was methylated in the edited founder larvae, while the *ywhaqa* on-target region was unmethylated (Fig. 6). Both for *sh2b3* and *ywhaqa*, the methylation patterns were nearly identical in the control sample. Similar results were obtained when examining 5mC methylation signals in the *ldlra* and *nbeal2* on-target regions (Additional file 1: Fig. S8). We therefore conclude that CRISPR-Cas9 genome editing has no significant effect on 5mC methylation patterns in our zebrafish experiment. This may be unsurprising since the Cas9 cleavage sites were located in coding exons. However, alterations in 5mC signals have been seen in experiments targeting differentially methylated regions of imprinted genomic loci [27], and it could occur also in other situations, e.g. when performing CRISPR-editing of promoters or regulatory elements. PureTarget enables linking the methylation pattern to a genome editing outcome on a single-molecule level. In addition, this approach can be used to assess the outcomes of DNA methylation editing [35].

## Discussion

PureTarget generated an average coverage of 1168x for the 30 successfully sequenced samples across seven on-target and off-target sites, and produced extremely accurate kilobase-length reads, with every read originating from a single molecule. These features make PureTarget an ideal method for verifying CRISPR-Cas9 induced small indels as well as larger SVs, allowing detection of genetic mosaicism down to allele frequencies of 1%.

We used a detection threshold of 1% VAF, although the > 1000x coverage generated by PureTarget would, in principle, be sufficient to identify alleles occurring at 0.5% or lower (Additional file 1: Fig. S7). We expect few, if any, false-positive variants at the 1% threshold, as no alternative alleles were detected in control DNA from uninjected wild-type zebrafish larvae. Detecting variants below 1% VAF, however, may require a more specialized laboratory setup to eliminate potential sources of contamination. The presence of low frequency mosaic variants may be important to consider in various applications of CRISPR–Cas9 genome editing, including breeding programs and the development of new therapies. However, determining what level of mosaicism should be considered acceptable is beyond the scope of this study. Such assessments should be performed on a case-by-case basis, following careful evaluation of the potential risks associated with each experiment.

Our comparison between PureTarget and LR-PCR showed striking differences in allele coverage. While LR-PCR often preferentially amplifies one of the alleles in heterozygous F1 fish, PureTarget gives an even representation of the two haplotypes. Further, PureTarget can be designed for longer than 5 kb regions, possibly up to 15–20 kb, which might be difficult to amplify by LR-PCR. The bias in LR-PCR data could partly be due to length differences between the two haplotypes, but in several cases, we found repeat elements in the target region to be inhibiting the amplification of one of the alleles. However, it should be noted that LR-PCR was performed using nanograms of DNA, which is a big advantage when sample material is limited, as compared to the micrograms of DNA required for amplification-free approaches. As suggested by a recent report, optimization of the amplification conditions or library preparation procedures could significantly improve the results of long-range amplicon sequencing [36]. For example, the LR-PCR results in our study would likely have improved by adding UMIs. However, UMI-based approaches are not perfect either, since errors may occur in the tag sequence [37]. Moreover, the UMIs can only remove duplicate molecules, but not other types of amplification biases. So, even though amplicon-based approaches can provide high multiplexing and cost-efficient sequencing, which may be preferable in some situations, we believe that an amplification-free enrichment protocol is better suited for analysis of genetic mosaicism.

In addition to PureTarget, amplification-free targeted sequencing can be performed using ONT adaptive sampling [15, 16], or ONT Cas9 enrichment [22, 23]. Each method has its own advantages and limitations. For PureTarget, the main limitation is the requirement to design two gRNAs flanking each target region to excise the ROIs. As a result, the method cannot detect events in which one of the gRNA binding sites is disrupted, for example by a structural variant or a large chromosomal aberration. Moreover, single nucleotide variants within a gRNA binding site or insertions larger than 10 kb in the ROI may cause allelic dropout. To ensure that all possible editing outcomes in a sample are captured, PureTarget may therefore need to be complemented with shallower whole-genome sequencing and/or additional gRNAs at each flank of the ROI for excision redundancy. In contrast, ONT adaptive sampling is based on in silico design of target regions, enabling detection of all variant types with no risk of allelic dropout. However, it provides limited enrichment [17] and therefore insufficient coverage for detailed analysis of genetic mosaicism. ONT’s Cas9 enrichment protocol, on the other hand, can generate deep coverage over target regions [27], and the allelic dropout issue can be mitigated by modifying the protocol to introduce only a single Cas9 cut [38]. But in any case, ONT sequencing currently lacks the ability of PureTarget to generate near-perfect reads. In this study, the single-molecule reads reached an average QV score of 39, corresponding to one error in approximately 8000 bases, and this level of accuracy is crucial for studying mosaic events occurring at low frequencies.

In this study, the CRISPR-Cas9 complex was injected into the fertilized zebrafish eggs at the single-cell stage. Our finding of up to 18 unique on-target editing events in a single founder zebrafish implies that CRISPR-Cas9 editing continues to take place after several cell divisions, as described elsewhere [28]. Interestingly, mosaicism was also present in the germ cells of founder zebrafish, based on the number of distinct editing events (*n* = 7 and *n* = 5) observed in DNA from pooled F1 zebrafish larvae and juvenile F1 zebrafish obtained from a founder pair. Our finding of widespread mosaicism in the germ cells of founder zebrafish is relevant for breeding programs where CRISPR-Cas9 is used. The results also raise further concerns for germline editing since it is difficult to predict which edits will segregate into future generations. However, it is possible that mosaicism can be mitigated for example by altering the CRISPR-Cas9 conditions, using other delivery systems, or more precise genome editing approaches like base editing [39].

In this experiment, 32 samples were multiplexed on a single Revio SMRTcell. This resulted in a relatively low cost per sample as compared with other amplification-free long-read sequencing methods. Given the large number of reads obtained, it is likely possible to further increase multiplexing and performance, e.g. by improving the target yield and accuracy of the HiFi reads. Enrichment may be optimized by altering the DNA input amount and Cas9 concentrations used in the PureTarget protocol and increased throughput could be achieved by modifying the conditions of loading the library on the Revio SMRTcell. Accuracy may be improved by increasing the movie acquisition time of the sequencing run. In this experiment the acquisition time was 24 h but it could be changed to 30 h to allow the molecules to be sequenced with even more passes. In addition to these methodological changes, perhaps the most important improvements will come from further developments in sequencing chemistry and long-read instrumentation. Lastly, the method can be implemented on the benchtop Vega instrument instead of the large-scale PacBio Revio, thereby reducing the capital investment cost and making the technology more accessible to individual laboratories.

## Conclusions

PureTarget gives a more detailed view than ever before into CRISPR-Cas9 induced genetic mosaicism. In this study, we applied the method to zebrafish samples, but it can be used equally well to verify genome-editing outcomes in other animal models, crop species, or genetically engineered cell samples developed for therapeutic applications. Although we did not see any effects of gene editing on methylation in the on-target regions, the feature of detecting 5mC patterns for individual DNA molecules can be highly relevant for example when studying the effects of editing in regulatory regions, or when assessing outcomes of DNA methylation editing. In summary, the ability to generate over 1000x target coverage of near-perfect native DNA reads opens up for a wide range of applications where it is necessary to study the full extent of genetic variation at specific genomic loci.

## Supplementary Information

Below is the link to the electronic supplementary material.

Supplementary Material 1: Additional file 1 – Supplementary figures and tables

Supplementary Material 2: Additional file 2 – SIQ file containing CRISPR-Cas9 editing events found in the PureTarget data.

## Data Availability

The datasets supporting the conclusions of this article are available in the European Nucleotide Archive (ENA) repository under accession number PRJEB110416, https://www.ebi.ac.uk/ena/browser/view/PRJEB110416 [40]. The genome editing outcomes for all 30 samples successfully sequenced with PureTarget and LR-PCR are available as a SIQ analysis spreadsheet result file (supplementary Data File S1). The file can be uploaded and viewed in the SIQplotteR website (https://siq.researchlumc.nl/SIQPlotteR/).

## Abbreviations

5mC: 5-Methylcytosine
BSA: Bovine serum albumin
CGU: Clinical Genomics Uppsala
dgRNPs: duplex guide RNPs
ENA: European Nucleotide Archive
GQN: Genomic Quality Number
gRNA: guide RNA
HiFi data: High accuracy PacBio data
IGV: Integrative Genomics Viewer
LR-PCR: Long-range PCR
NAISS: National Academic Infrastructure for Supercomputing in Sweden
NGI: National Genomics Infrastructure
ONT: Oxford Nanopore Technologies
PacBio: Pacific Biosciences
ROI: Region of interest
SIQ: Sequence interrogation and qualification software
SV: Structural variant
UMIs: Unique molecular identifiers
VAF: Variant allele frequency
WG-LRS: Whole genome long-read sequencing
