# Supplementary material for: Accurate characterization of CRISPR-Cas9 genome editing outcomes and mosaicism with near-perfect long reads
Source: Genome Med. 2026 Sep 15;18:135. doi: 10.1186/s13073-026-01772-1 (PMC13587479; doi:10.1186/s13073-026-01772-1)
Supplement: Supplementary file 1 [file 13073_2026_1772_MOESM1_ESM.pdf]

Supplementary Figures

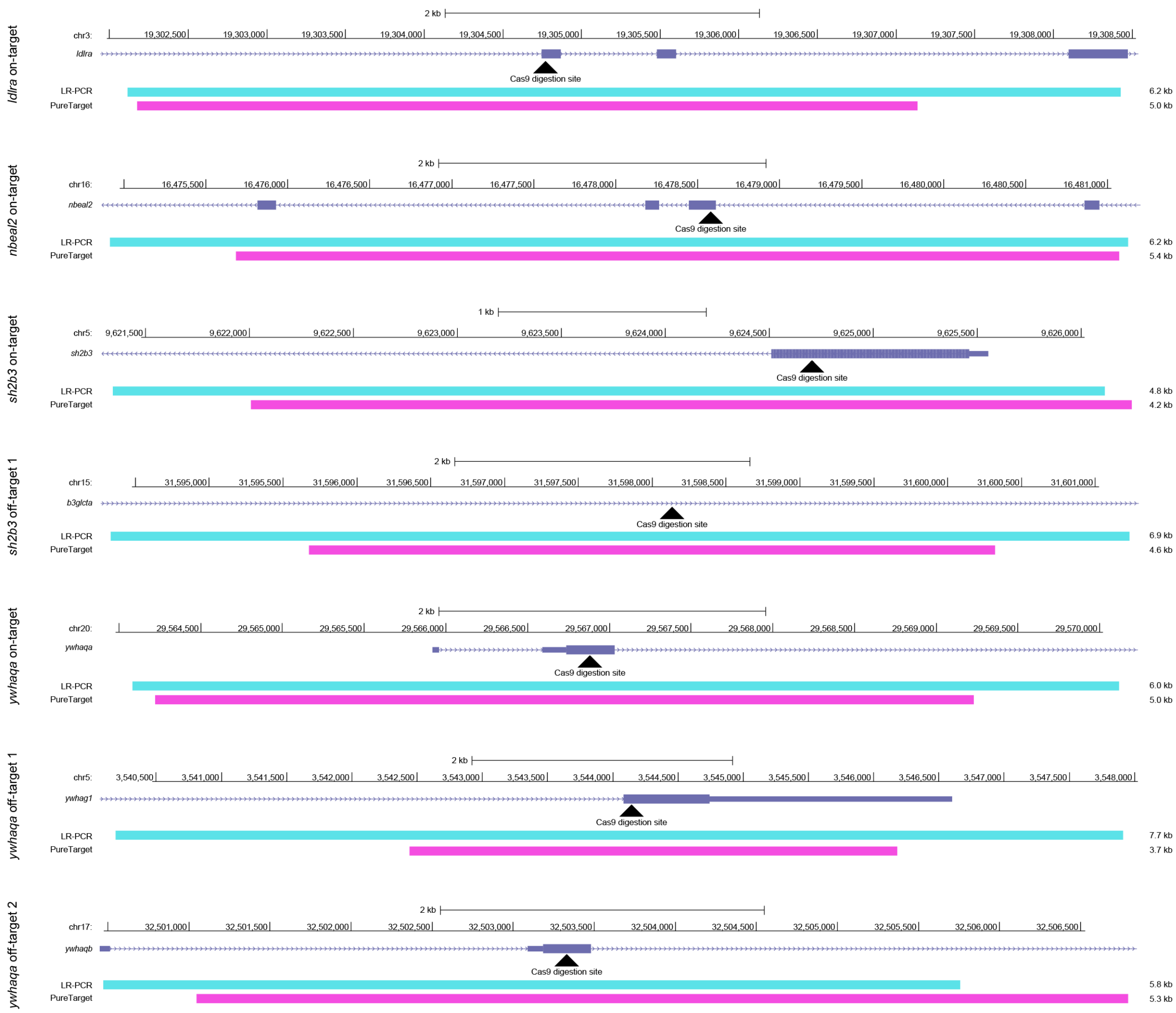

Fig. S1. LR-PCR and PureTarget design. Design overview and comparison of the region of interests (ROIs) for LR-PCR (turquoise) and PureTarget (magenta) for all seven targets. The black arrow marks the predicted Cas9 digestion site from the genome editing experiment for each target.

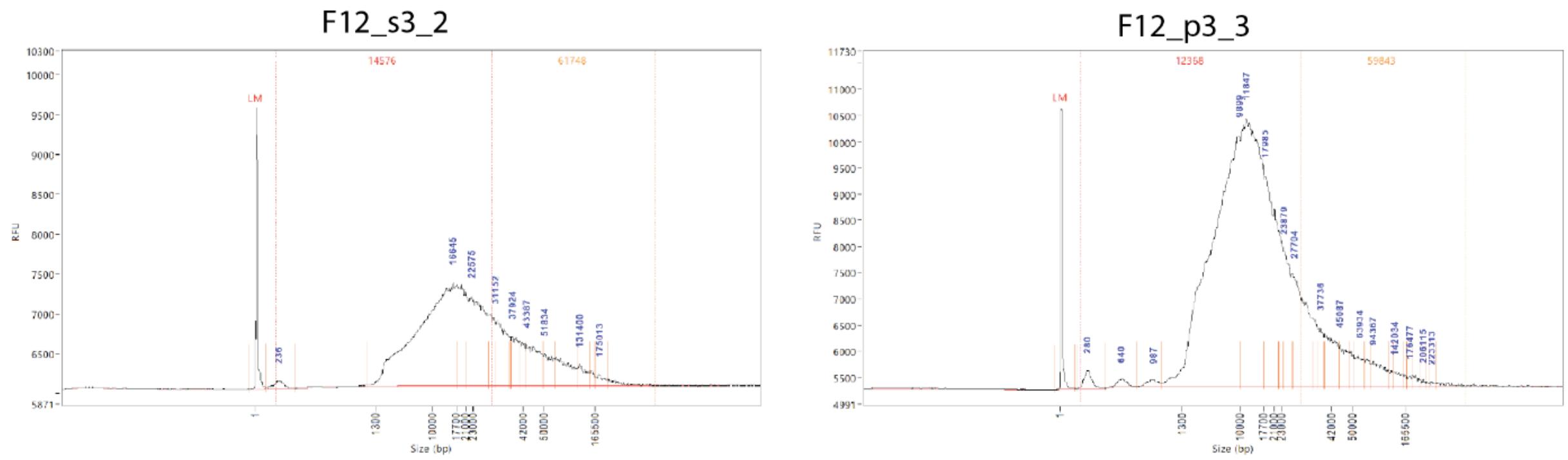

Fig. S2. Quality of zebrafish DNA. Examples of DNA integrity of the zebrafish DNA samples. The DNA integrity was assessed using the Agilent FemtoPulse system and the Genomic DNA 165 kb kit. The sample to the left is DNA extracted from an individual zebrafish and the sample to the right is DNA extracted from a pool of zebrafish larvae. Genomic Quality Numbers (GQN) at 30kb, for all samples, is available in supplementary Table S4.

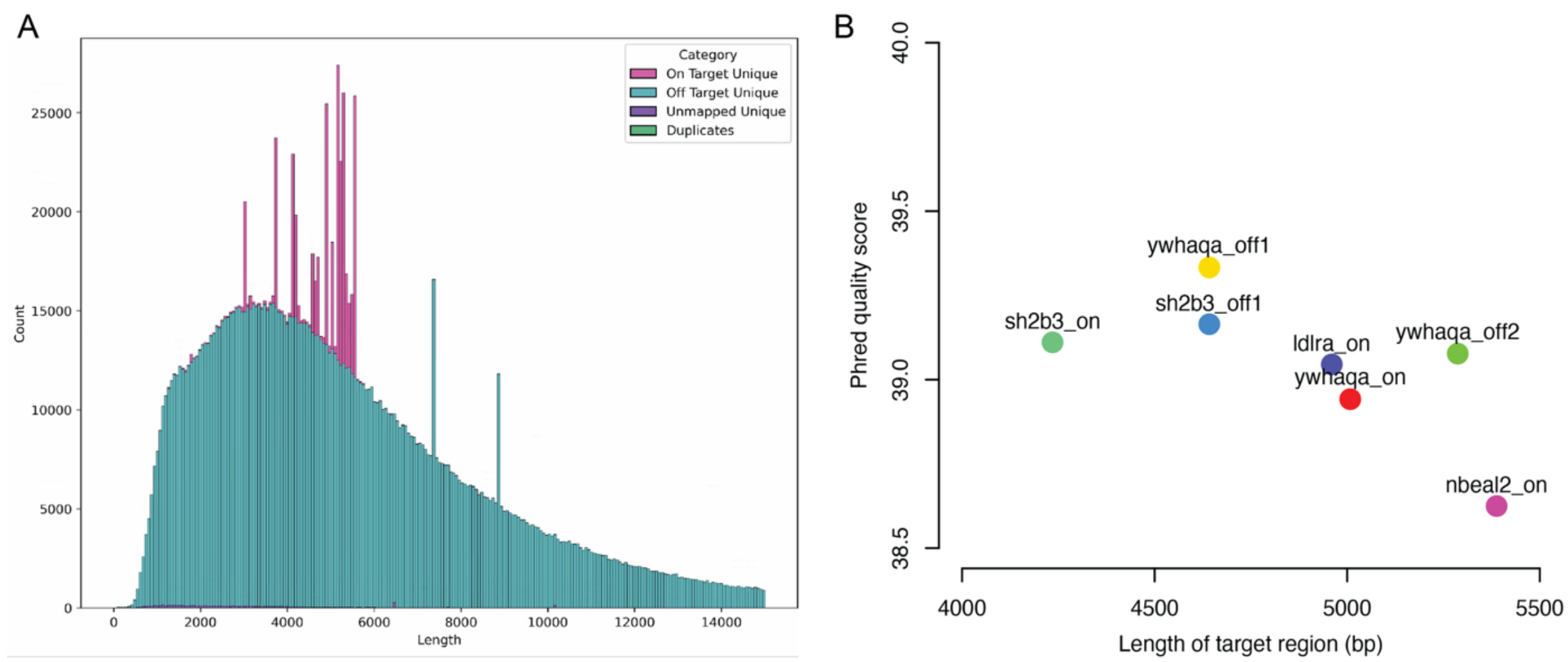

Fig. S3. Read length and quality of PureTarget sequencing. A) Read length distribution of the PureTarget sequencing run. The pink bars correspond to reads that map to the target regions. **B)** Distribution of quality scores for PureTarget on-target reads. On the y-axis are the average phred quality scores for all reads mapping to the seven targets in the PureTarget panel. All seven targets have phred scores between 38.5 and 39.5, with slightly higher scores for shorter target regions.

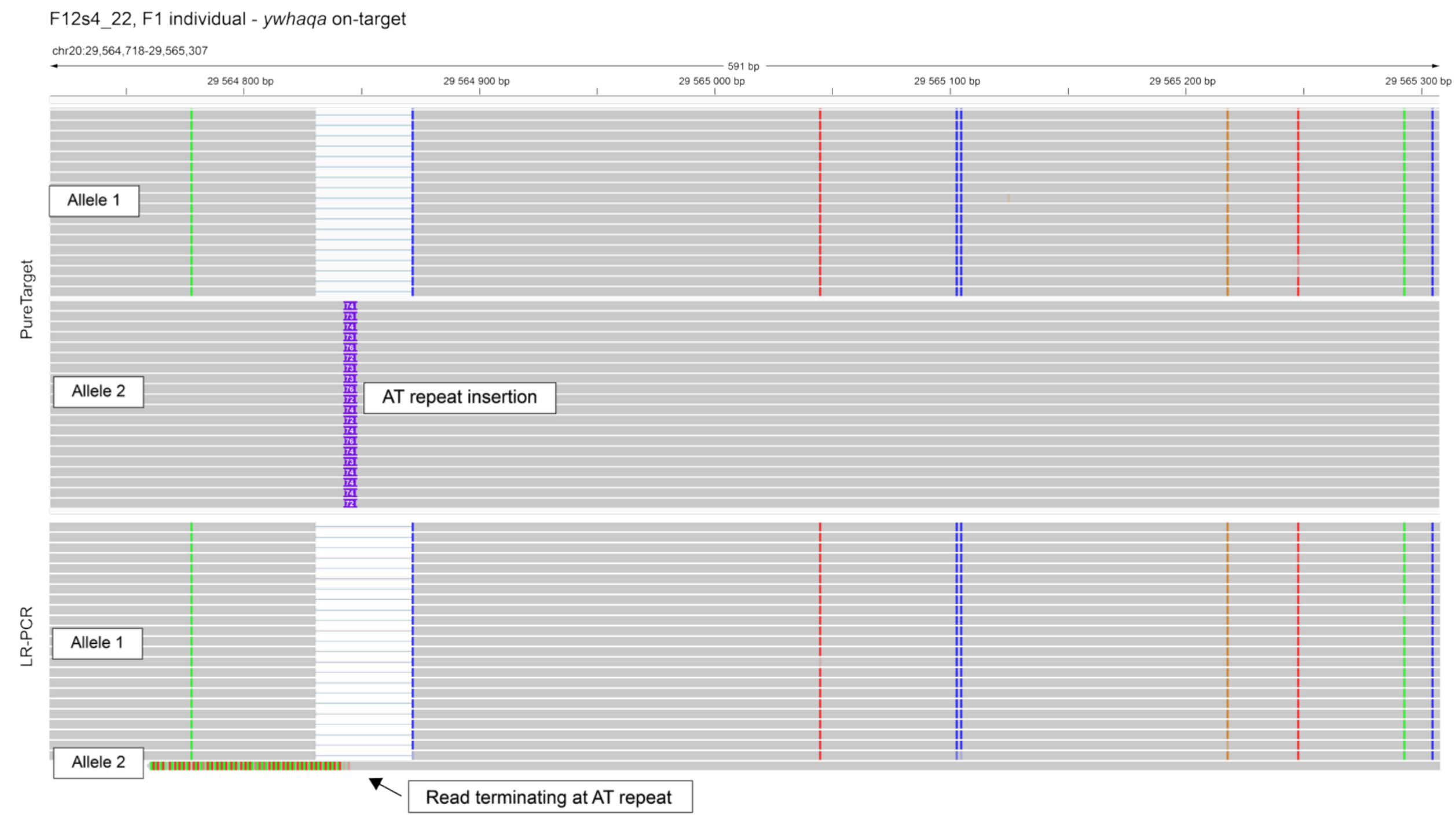

Fig. S4. Example of AT repeat causing amplification bias. IGV image showing the two different alleles at *ywhaqa* on-target for sample F1 individual sample F12s4_22. For each allele and data type, up to 25 representative PacBio HiFi reads are shown. In the PureTarget data, each allele is represented by around 50% of the reads. Allele 1 carries a deletion, while an AT repeat of approximately 74 bp is present in allele 2. There is a small variation on repeat length between individual molecules, but the 74 bp repeat is the most abundant. In the LR-PCR data, only one read represent allele 2 and it is terminating at the site of the 74 bp AT repeat.

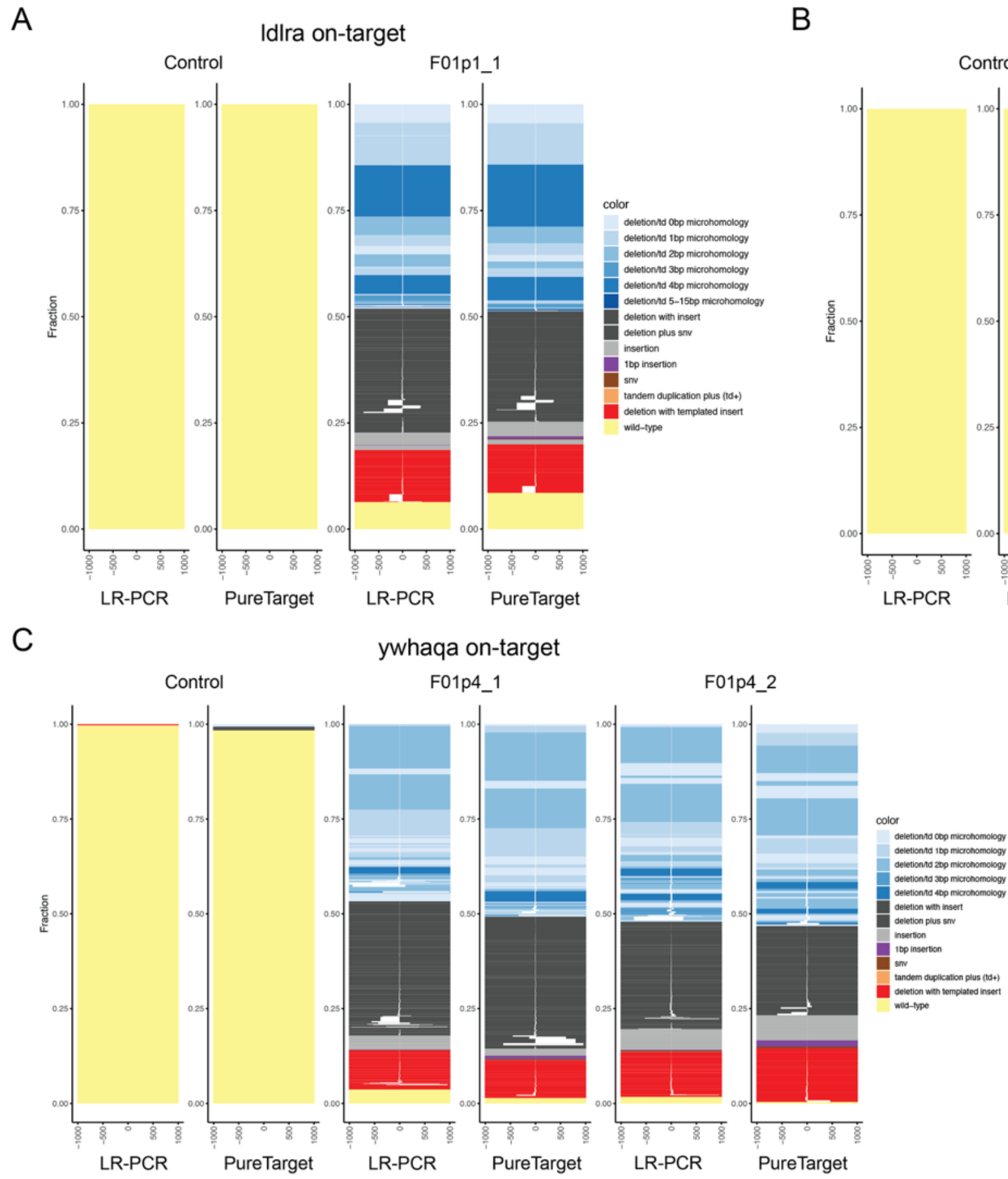

Fig. S5. CRISPR-Cas9 induced genetic mosaicism at on-target sites for pools of founder zebrafish larvae. A) SIQ tornado plots showing the distribution of on-target genome editing outcomes in the uninjected control sample and a pool of *ldlra* founder (F0) larvae. For each sample, outcomes are shown both for LR-PCR and PureTarget data in a window centered around the CRISPR-Cas9 editing site. **B)** SIQ tornado plots for an uninjected control sample and a pool of *nbeal2* founder (F0) larvae. **C)** SIQ tornado plots for an uninjected control sample and two pools of *ywhaqa* founder (F0) larvae.

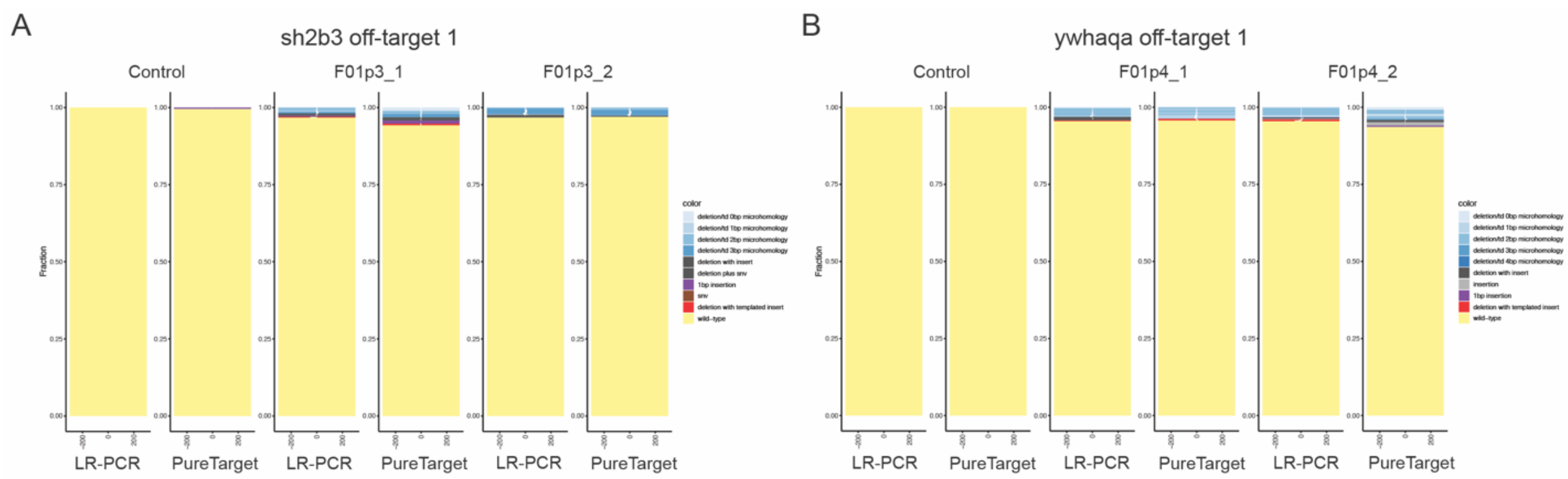

Fig. S6. CRISPR-Cas9 induced genetic mosaicism at off-target sites for pools of founder zebrafish larvae. A) SIQ tornado plots showing the distribution of off-target genome editing outcomes at *sh2b3* off-target site 1 in the uninjected control sample and two pools of *sh2b3* founder (F0) larvae. For each sample, outcomes are shown both for LR-PCR and PureTarget data in a window centered around the CRISPR-Cas9 editing site. **B)** SIQ tornado plots showing the distribution of off-target genome editing outcomes for an uninjected control sample and two pools of *ywhaqa* founder (F0) larvae at *ywhaqa* off-target site 1.

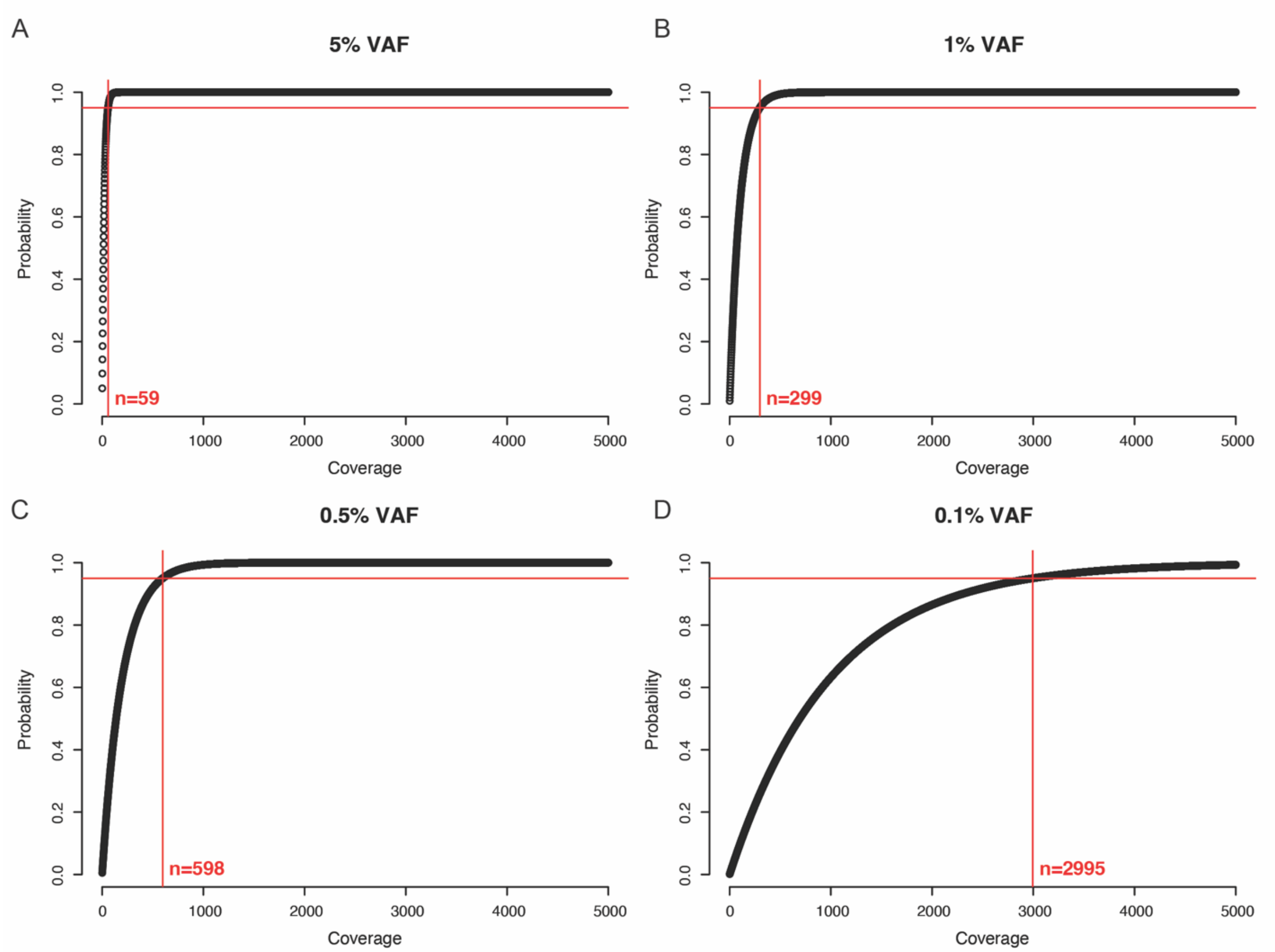

Fig. S7. Coverage required to detect mosaicism at different variant allele frequency (VAF) levels. The plots show the theoretical coverage required to find a mosaic variant at a 95% confidence level at 5%, 1%, 0.5% and 0.1% VAF. The calculations are based on the assumptions that i) the reads contain no sequencing errors and ii) one single read containing a mosaic variant is sufficient for detection. Under these assumptions the probability of detecting the mosaic variant follows the distribution $p(x,n)=1-(1-x)^n$, where x is the VAF level and n is the coverage. The black dots show the value of p(x,n) at coverage levels ranging between 1 and 5000. The red horizontal line shows the 95% probability, and the vertical red line represents the coverage required to reach that confidence level. The results show that A) n=59 is required for detection of a 5% VAF, B) n=299 for 1% VAF, C) n=598 for 0.5% VAF, and D) n=2995 for 0.1% VAF.

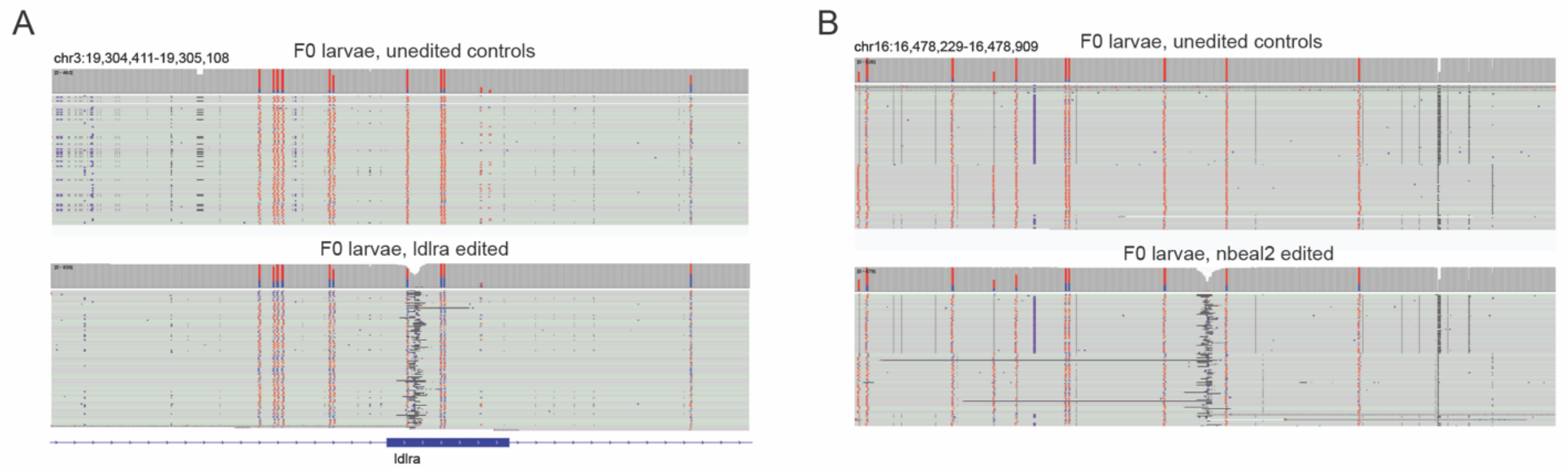

Fig. S8. Comparison of 5mC CpG methylation signals between CRISPR-Cas9 edited and control samples. A) IGV view showing 5mC signals in the PureTarget data from a pool of *ldlra* founder larvae (bottom) compared with a sample of pooled unedited controls (top). **B)** Methylation signals in a pool of *nbeal2* founder larvae (bottom) compared with a sample of pooled controls (top).

Supplementary Tables

Table S1: Study cohort information

Sample ID	Gene targeted	Generation	Age	Fish per sample	Tissue collected	Analyses performed
F01p1_1	*ldlra*	F0	10 dpf	30	whole larvae	LR-PCR, PureTarget
F01p2_1	*nbeal2*	F0	10 dpf	25	whole larvae	LR-PCR, PureTarget
F01p3_1	*sh2b3*	F0	10 dpf	30	whole larvae	LR-PCR, PureTarget
F01p3_2	*sh2b3*	F0	10 dpf	30	whole larvae	PureTarget
F01p4_1	*ywhaqa*	F0	10 dpf	30	whole larvae	LR-PCR, PureTarget
F01p4_2	*ywhaqa*	F0	10 dpf	30	whole larvae	LR-PCR, PureTarget
F01s3_2	*sh2b3*	F0	adult	1	whole fish	LR-PCR, PureTarget
F01s3_3	*sh2b3*	F0	adult	1	whole fish	LR-PCR, PureTarget
F01s3_4	*sh2b3*	F0	adult	1	whole fish	LR-PCR, PureTarget
F01s3_5	*sh2b3*	F0	adult	1	whole fish	LR-PCR, PureTarget
F01s3_6	*sh2b3*	F0	adult	1	whole fish	LR-PCR, PureTarget
F01s4_3	*ywhaqa*	F0	adult	1	whole fish	LR-PCR, PureTarget
F01s4_4	*ywhaqa*	F0	adult	1	whole fish	LR-PCR, PureTarget
F01s4_6	*ywhaqa*	F0	adult	1	whole fish	LR-PCR, PureTarget
F01s4_15	*ywhaqa*	F0	adult	1	whole fish	LR-PCR, PureTarget
F01s4_16	*ywhaqa*	F0	adult	1	whole fish	LR-PCR, PureTarget
F12p3_3	*sh2b3*	F1	5 dpf	30	whole larvae	LR-PCR, PureTarget
F12p3_4	*sh2b3*	F1	5 dpf	30	whole larvae	LR-PCR, PureTarget
F12p3_6	*sh2b3*	F1	5 dpf	30	whole larvae	LR-PCR, PureTarget
F12p4_4	*ywhaqa*	F1	5 dpf	30	whole larvae	LR-PCR, PureTarget
F12p4_5	*ywhaqa*	F1	5 dpf	30	whole larvae	LR-PCR, PureTarget
F12p4_6	*ywhaqa*	F1	5 dpf	30	whole larvae	LR-PCR, PureTarget
F12s3_2	*sh2b3*	F1	juvenile	1	fin clip	LR-PCR, PureTarget
F12s3_3	*sh2b3*	F1	juvenile	1	fin clip	LR-PCR, PureTarget
F12s3_12	*sh2b3*	F1	juvenile	1	fin clip	LR-PCR, PureTarget
F12s3_13	*sh2b3*	F1	juvenile	1	fin clip	LR-PCR, PureTarget
F12s3_14	*sh2b3*	F1	juvenile	1	fin clip	LR-PCR, PureTarget
F12s4_20	*ywhaqa*	F1	juvenile	1	fin clip	LR-PCR, PureTarget
F12s4_22	*ywhaqa*	F1	juvenile	1	fin clip	LR-PCR, PureTarget
F12s4_23	*ywhaqa*	F1	juvenile	1	fin clip	LR-PCR, PureTarget
F12s4_24	*ywhaqa*	F1	juvenile	1	fin clip	LR-PCR, PureTarget
F01wt_01	uninjected	F0	10 dpf	30	whole larvae	LR-PCR, PureTarget

Table S2: Information on guide RNAs used in genome editing of zebrafish eggs

gRNA name	Targeted sequence	gRNA sequence	Genomic coordinates
ldlra	GATTCACGGCAGTATCAGTG	GATTCACGGCAGTATCAGTG	chr3:19304761-19304780
nbeal2	TGTCCCCCTTCTCCCCAAGG	GGTCCCCCTTCTCCCCAAGG	chr16:16478569-16478588
sh2b3	GGGAGCGCTGGAATCCATGG	GGGAGCGCTGGAATCCATGG	chr5:9624695-9624714
ywhaqa	GTCGCCTACAAGAACGTGGT	GGCGCCTACAAGAACGTGGT	chr20:29566873-29566892

Table S3: Primer information used for LR-PCRs

Primer ID	Primer sequence	Genomic coordinates	ROI size (bp)
ldlra_on_fwd	GGACTGATCTGCCTCTGAATG	chr3:19302116-19302136	6290
ldlra_on_rev	CACTTCCAGCTACCGTGTATG	chr3:19308406-19308426	
nbeal2_on_fwd	GAGATGGGTGAATGGAAGGATAA	chr16:16474916-16474938	6211
nbeal2_on_rev	TCTTGGATGACCTAAGGGTAGA	chr16:16481105-16481126	
sh2b3_on_fwd	GAGATCCGAACTGCTGGATTTA	chr5:9621342-9621363	4771
sh2b3_on_rev	GTTTGGCTTGCTGAGCTTATG	chr5:9626092-9626112	
sh2b3_off1_fwd	CTCTTGCTATTTCGGGCTAGTG	chr15:31594333-31594354	6898
sh2b3_off1_rev	GTGTCGTGATGTCTCTGTCTTG	chr15:31601209-31601230	
ywhaqa_on_fwd	CTCTTTCCGCTCCCTATGAATG	chr20:29564081-29564102	6038
ywhaqa_on_rev	GTGATGAGGTGCCAAGCTATAA	chr20:29570097-29570118	
ywhaqa_off1_fwd	TTCACGCTACCTCTGTTCTTG	chr5:3540188-3540208	7703
ywhaqa_off1_rev	CGTGCAGCCCTATAGTTCTAAT	chr5:3547891-3547912	
ywhaqa_off2_fwd	CATCGGTGTGCTAAACTGAAATG	chr17:32501047-32501069	5746
ywhaqa_off2_rev	GTCTCTCTGCGAACAAGGTAAA	chr17:32506771-32506792	

Table S4: Information on PureTarget gRNAs and regions of interest (ROIs)

gRNA ID	Strand	Sequence	PAM	Genomic coordinates	ROI size (bp)
ldlra_on_fwd	+	TAATAATTTTCTACGTCCTA	CGG	chr3:19302176-19302195	4961
ldlra_on_rev	-	GTCAACATAGAAATTTCAGA	TGG	chr3:19307117-19307136	
nbeal2_on_fwd	+	AGAGCTGAAAGGTAAGGACT	GGG	chr16:16475683-16475702	5389
nbeal2_on_rev	-	TAGTTCATGACCTAGACCTC	AGG	chr16:16481052-16481071	
sh2b3_off1_fwd	+	AACCACATAACCTTTTCTCC	CGG	chr15:31595678-31595697	4643
sh2b3_off1_rev	-	AATTCCTCAATTCTAGGATA	GGG	chr15:31600301-31600320	
sh2b3_on_fwd	+	TTAAAGCCAACTGGTTCCCA	CGG	chr5:9622006-9622025	4236
sh2b3_on_rev	-	GATATTCACCACACCTCAGT	TGG	chr5:9626222-9626241	
ywhaqa_off1_fwd	+	CGGATTGGGCAAATGCCAGA	AGG	chr5:3542442-3542461	3739
ywhaqa_off1_rev	-	ATGGTAACCACGCACTGTTT	TGG	chr5:3546161-3546180	
ywhaqa_off2_fwd	+	CTCAAAGGCTATCAATCCGG	TGG	chr17:32500471-32500490	5288
ywhaqa_off2_rev	-	ACTAGAGTGCTGACAATGAT	GGG	chr17:32505739-32505758	
ywhaqa_on_fwd	+	TAGATAAGCCAAGTAGACTC	TGG	chr20:29564222-29564241	5009
ywhaqa_on_rev	-	CTACTTTAAAGGTGCCCAGA	AGG	chr20:29569211-29569230	

Table S5: Characteristics of all 32 zebrafish DNA samples run with PureTarget

Sample name	Generation	Edit	Age	Mating pair	Sample ID	GQN 30 kb
F12p3_3	F1	*sh2b3*	5 dpf 30 larvae	sh2b3 pair 3	pr_180_001	2,8
F12p3_4	F1	*sh2b3*	5 dpf 30 larvae	sh2b3 pair 3	pr_180_002	3,5
F12p3_6	F1	*sh2b3*	5 dpf 30 larvae	sh2b3 pair 3	pr_180_003	3,4
F12s3_2	F1	*sh2b3*	Juvenile fin	sh2b3 pair 3	pr_180_004	1,4
F12s3_3	F1	*sh2b3*	Juvenile fin	sh2b3 pair 3	pr_180_005	1,1
F12s3_12	F1	*sh2b3*	Juvenile fin	sh2b3 pair 3	pr_180_006	3
F12s3_13	F1	*sh2b3*	Juvenile fin	sh2b3 pair 3	pr_180_007	3,9
F12s3_14	F1	*sh2b3*	Juvenile fin	sh2b3 pair 3	pr_180_008	4,9
F12p4_4	F1	*ywhaqa*	5 dpf 30 larvae	ywhaqa pair 2	pr_180_009	2,7
F12p4_5	F1	*ywhaqa*	5 dpf 30 larvae	ywhaqa pair 2	pr_180_010	4,3
F12p4_6	F1	*ywhaqa*	5 dpf 30 larvae	ywhaqa pair 2	pr_180_011	5,7
F12s4_20	F1	*ywhaqa*	Juvenile fin	ywhaqa pair 2	pr_180_012	7
F12s4_22	F1	*ywhaqa*	Juvenile fin	ywhaqa pair 2	pr_180_013	6,6
F12s4_23	F1	*ywhaqa*	Juvenile fin	ywhaqa pair 2	pr_180_014	5,8
F12s4_24	F1	*ywhaqa*	Juvenile fin	ywhaqa pair 2	pr_180_015	7,1
F01wt_1	F0	*wt*	10 dpf 30 larvae		pr_180_016	4
F01p3_1	F0	*sh2b3*	10 dpf 30 larvae		pr_180_017	1,8
F01p3_2	F0	*sh2b3*	10 dpf 30 larvae		pr_180_018	2,1
F01p4_1	F0	*ywhaqa*	10 dpf 30 larvae		pr_180_019	2,2
F01p4_2	F0	*ywhaqa*	10 dpf 30 larvae		pr_180_020	1,9
F01p1_1	F0	*ldlra*	10 dpf 30 larvae		pr_180_021	3,6
F01p2_1	F0	*nbeal2*	10 dpf 25 larvae		pr_180_022	2,8
F01s3_2	F0	*sh2b3*	adult		pr_180_023	0,6
F01s3_3*	F0	*sh2b3*	adult		pr_180_024	0
F01s3_4	F0	*sh2b3*	adult		pr_180_025	0
F01s3_5*	F0	*sh2b3*	adult		pr_180_026	0,4
F01s3_6	F0	*sh2b3*	adult		pr_180_027	0,2
F01s4_3	F0	*ywhaqa*	adult		pr_180_028	3,7
F01s4_4	F0	*ywhaqa*	adult		pr_180_029	3,9
F01s4_6	F0	*ywhaqa*	adult		pr_180_030	1,9
F01s4_15	F0	*ywhaqa*	adult		pr_180_031	2
F01s4_16	F0	*ywhaqa*	adult		pr_180_032	1,7

*These two samples failed to generate 30X coverage over the target sites and were excluded from the analysis

Table S6: PureTarget run statistics for one Revio 8M SMRT cell

HiFi Bases	10,377,051,654
HiFi Reads	1,826,314
Median HiFi Read Length (bp)	4,925
Median HiFi Read Quality	QV41
Sample count	32
Target regions	7
Total Target Length (bp)	33,258
Percent of bases mapping to target regions (min-max for the 32 samples)	7.1% - 18.4%

*Results obtained by running the Target Enrichment plugin in SMRTLink v.25.2

Table S7: Number of PureTarget reads mapping to the seven target sites

Sample	*ywhaqa* on1	*ywhaqa* off1	*ywhaqa* off2	*sh2b3* on	*sh2b3* off1	*ldlra* on	*nbeal2* on	all target reads	total reads	% on target
F12p3_3	1314	1842	2686	4602	1416	1238	2138	15236	83948	18.1
F12p3_4	1364	1812	2940	4996	1376	1148	2302	15938	79722	20
F12p3_6	1548	1954	2980	4844	1472	1482	2216	16496	74211	22.2
F12s3_2	698	1326	1204	3146	860	908	1110	9252	82765	11.2
F12s3_3	448	756	940	2640	614	698	880	6976	72641	9.6
F12s3_12	880	1444	1978	3138	858	988	1780	11066	55056	20.1
F12s3_13	716	1250	1446	3400	698	804	1212	9526	47696	20
F12s3_14	666	736	1168	2704	658	432	1056	7420	34783	21.3
F12p4_4	1364	1928	2904	4814	1434	1802	2528	16774	57986	28.9
F12p4_5	1286	1690	2354	3826	1252	1638	2204	14250	43903	32.5
F12p4_6	1128	1784	2650	4012	1262	1548	2122	14506	46441	31.2
F12s4_20	584	946	1566	3020	618	976	1302	9012	40312	22.4
F12s4_22	460	818	1222	1648	406	802	992	6348	19183	33.1
F12s4_23	1224	2310	2032	5942	1218	1968	2288	16982	30532	55.6
F12s4_24	616	1166	1346	2284	666	778	1152	8008	27441	29.2
F01wt_1	560	1036	1606	1700	764	950	1278	7894	49305	16
F01p3_1	676	1634	1736	3034	846	1268	1584	10778	100440	10.7
F01p3_2	842	2122	2142	4014	1166	1602	2028	13916	122110	11.4
F01p4_1	956	1994	2328	3536	1156	1510	1964	13444	81800	16.4
F01p4_2	630	1452	1786	3030	804	1024	1584	10310	72999	14.1
F01p1_1	978	1738	2658	3928	1196	1760	2250	14508	71502	20.3
F01p2_1	572	1202	1492	2526	796	1234	1472	9294	60767	15.3
F01s3_2	300	930	622	1556	420	500	552	4880	56562	8.6
F01s3_3*	2	2	4	2	0	0	2	12	5633	0.2
F01s3_4	70	224	150	176	108	124	148	1000	31796	3.1
F01s3_5*	6	64	16	14	8	8	4	120	20914	0.6
F01s3_6	378	928	554	1002	378	474	466	4180	60724	6.9
F01s4_3	940	2696	3166	5888	156	2090	2602	17538	62493	28.1
F01s4_4	286	624	624	838	22	470	412	3276	25010	13.1
F01s4_6	1020	1616	2402	3862	1234	1744	2040	13918	110901	12.5
F01s4_15	404	570	732	604	480	454	664	3908	48161	8.1
F01s4_16	216	754	606	746	142	370	426	3260	38142	8.5
								310026	1815879	17.1

*These two samples failed to generate 30X coverage over the target sites and were excluded from the analysis

Table S8: Allelic distribution of reads at heterozygous SNVs in F1 fish

Sample	Method	Target	SNV position* (ref/alt)	Total	Ref	Alt
F12s3_2	LR-PCR	sh2b3 on	chr5:9624430 (C/A)	1102	372 (34%)	729 (66%)
F12s3_2	PureTarget	sh2b3 on	chr5:9624430 (C/A)	1582	762 (48%)	820 (52%)
F12s3_12	LR-PCR	sh2b3 on	chr5:9624430 (C/A)	1402	177 (13%)	1225 (87%)
F12s3_12	PureTarget	sh2b3 on	chr5:9624430 (C/A)	2098	1074 (51%)	1024 (49%)
F12s3_13	LR-PCR	sh2b3 on	chr5:9624430 (C/A)	1396	109 (8%)	1287 (92%)
F12s3_13	PureTarget	sh2b3 on	chr5:9624430 (C/A)	1702	890 (52%)	812 (48%)
F12s4_20	LR-PCR	ywhaqa on	chr20:29566589 (T/C)	1597	1 (0%)	1595 (100%)
F12s4_20	PureTarget	ywhaqa on	chr20:29566589 (T/C)	574	272 (47%)	302 (53%)
F12s4_20	LR-PCR	ywhaqa off2	chr17:32502859 (T/C)	2911	282 (10%)	2629 (90%)
F12s4_20	PureTarget	ywhaqa off2	chr17:32502859 (T/C)	1556	688 (44%)	868 (56%)
F12s4_22	LR-PCR	ywhaqa on	chr20:29566589 (T/C)	1567	3 (0%)	1563 (100%)
F12s4_22	PureTarget	ywhaqa on	chr20:29566589 (T/C)	456	234 (51%)	222 (49%)
F12s4_22	LR-PCR	ywhaqa off1	chr5:3544380 (C/T)	1539	1287 (84%)	252 (16%)
F12s4_22	PureTarget	ywhaqa off1	chr5:3544380 (C/T)	818	378 (46%)	440 (54%)
F12s4_22	LR-PCR	ywhaqa off2	chr17:32502859 (T/C)	4273	479 (11%)	3793 (89%)
F12s4_22	PureTarget	ywhaqa off2	chr17:32502859 (T/C)	1218	608 (50%)	610 (50%)
F12s4_23	LR-PCR	ywhaqa on	chr20:29566589 (T/C)	1164	18 (2%)	1145 (98%)
F12s4_23	PureTarget	ywhaqa on	chr20:29566589 (T/C)	1212	608 (50%)	604 (50%)
F12s4_23	LR-PCR	ywhaqa off1	chr5:3543062 (C/T)	1000	729 (73%)	269 (27%)
F12s4_23	PureTarget	ywhaqa off1	chr5:3543062 (C/T)	2304	1136 (49%)	1168 (51%)
F12s4_24	LR-PCR	ywhaqa off1	chr5:3543062 (C/T)	1765	1374 (78%)	389 (22%)
F12s4_24	PureTarget	ywhaqa off1	chr5:3543062 (C/T)	1162	546 (47%)	616 (53%)

*For each LR-PCR amplicon, a representative SNV position was selected for calculating the allele frequency. The SNVs were selected to be located outside copy number variable regions and gaps in the GRCz11 reference.
